# Coordination of Chromatid Separation and Spindle Elongation by Antagonistic Activities of Mitotic and S-Phase CDKs

**DOI:** 10.1371/journal.pgen.1003319

**Published:** 2013-02-28

**Authors:** Fengshan Liang, Daniel Richmond, Yanchang Wang

**Affiliations:** Department of Biomedical Sciences, College of Medicine, Florida State University, Tallahassee, Florida, United States of America; The University of North Carolina at Chapel Hill, United States of America

## Abstract

Because cohesion prevents sister-chromatid separation and spindle elongation, cohesion dissolution may trigger these two events simultaneously. However, the relatively normal spindle elongation kinetics in yeast cohesin mutants indicates an additional mechanism for the temporal control of spindle elongation. Here we show evidence indicating that S-phase CDK (cyclin dependent kinase) negatively regulates spindle elongation. In contrast, mitotic CDK promotes spindle elongation by activating Cdc14 phosphatase, which reverses the protein phosphorylation imposed by S-phase CDK. Our data suggest that S-phase CDK negatively regulates spindle elongation partly through its phosphorylation of a spindle pole body (SPB) protein Spc110. We also show that hyperactive S-phase CDK compromises the microtubule localization of Stu2, a processive microtubule polymerase essential for spindle elongation. Strikingly, we found that hyperactive mitotic CDK induces uncoupled spindle elongation and sister-chromatid separation in securin mutants (*pds1*Δ), and we speculate that asynchronous chromosome segregation in *pds1*Δ cells contributes to this phenotype. Therefore, the tight temporal control of spindle elongation and cohesin cleavage assure orchestrated chromosome separation and spindle elongation.

## Author Summary

Before anaphase onset, the presence of securin Pds1 prevents the cleavage of cohesin, a protein complex that holds sister chromatids together, but the degradation of Pds1 leads to robust cohesion dissolution, resulting in synchronous separation of all chromosomes. Since sister chromatids are attached to spindle microtubules before anaphase onset, spindle elongation segregates sister chromatids to each daughter cell after cohesion dissolution. A fundamental question is how spindle elongation is temporally regulated in order to coordinate with sister chromatid separation. Cyclin-dependent kinases (CDKs) play a key role in the cell cycle, and it is well established that S-phase CDK promotes DNA synthesis. We found that hyperactive S-phase CDK delays spindle elongation in budding yeast, while hyperactive mitotic CDK leads to premature spindle elongation. We further provide evidence indicating that mitotic CDK promotes spindle elongation by activating a protein phosphatase that antagonizes S-phase CDK, thus the balance of S-phase and mitotic CDK controls the timing of spindle elongation. Interestingly, hyperactive mitotic CDK induces uncoupled spindle elongation and sister chromatid separation in the absence of securin, suggesting that the temporal control of spindle elongation and cohesin cleavage is essential for faithful chromosome segregation.

## Introduction

In eukaryotic cells, spindle elongation and sister-chromatid separation are two critical mitotic events, and the coordination of these two events is essential for the fidelity of chromosome segregation. During mitosis, the cleavage of sister chromatid cohesion allows the sister chromatids to move toward the respective spindle pole, then the spindle elongates further to pull sister chromatids into two daughter cells [1]. Because sister-chromatid cohesion prevents sister chromatid separation and spindle elongation, these two events could be coupled by the cleavage of cohesin. Before anaphase entry, cohesin cleavage is prohibited by the presence of securin (Pds1), which binds to and inhibits separase (Esp1) that cleaves cohesin Scc1/Mcd1 [2], [3]. The degradation of Pds1 before anaphase entry alleviates the inhibition of Esp1, resulting in the robust cohesin cleavage and simultaneous sister-chromatid separation [2], [4], [5]. However, yeast cells lacking either Pds1 or cohesin do not show premature spindle elongation, indicating that a cohesion-independent mechanism controls the timing of spindle elongation [6].

CDKs are the key driving force for cell cycle progression. In budding yeast, S-phase cyclins Clb5 and Clb6 appear during S-phase, which is consistent with their function in DNA synthesis. Compared to mitotic CDK Clb2-Cdk1, Clb5-Cdk1 shows stronger kinase activity toward a subset of CDK substrates [7]. In addition to proteins involved in DNA synthesis, other Clb5-specific substrates, such as Sli15, Fin1, Ase1, and Spc110, associate with the SPB or microtubules, indicating that S-phase CDK may also regulate spindle dynamics [8]–[10].

The tightly regulated activity of CDK and phosphatase enables unique temporal phosphorylation kinetics of each CDK substrate during the cell cycle. The periodical expression of cyclins controls the activity and substrate specificity of the CDK, while a conserved protein phosphatase Cdc14 reverses the phosphorylation of these CDK substrates [11]. In budding yeast, the regulation of Cdc14 activity is achieved through its subcellular localization. Before anaphase entry, Cdc14 is sequestered within the nucleolus by binding to a nucleolar protein Net1 [12], [13]. The dephosphorylation of Net1 by PP2A^Cdc55^ ensures a strong Net1-Cdc14 interaction, while during early anaphase the phosphorylation of Net1 by mitotic CDK, Clb2-Cdk1, triggers the release of Cdc14 from the nucleolus [14]–[17]. The degradation of Pds1 frees separase Esp1, which not only cleaves cohesin rings but also down-regulates PP2A^Cdc55^ with the assistance of other FEAR components [15]. The increased ratio of Clb2-Cdk1/PP2A^Cdc55^ leads to Net1 phosphorylation and the subsequent Cdc14 release. Because S-phase cyclin Clb5 is also degraded before anaphase entry [18], the combination of increased Cdc14 and decreased Clb5-Cdk1 activity during early anaphase results in the dephosphorylation of Clb5-specific substrates [19], [20]. Therefore, mitotic CDK may promote mitotic progression by reversing the phosphorylation imposed by S-phase CDK.

Recent work in the Amon lab has shown that the loss of function of both mitotic cyclins Clb1 and Clb2 in *clb1*Δ *clb2-IV* mutant cells prevents spindle elongation, indicating the essential role of mitotic CDK in this process. Moreover, neither the loss of sister-chromatid cohesion nor deletion of securin Pds1 is able to rescue the spindle elongation defect in *clb1*Δ *clb2-IV* mutant cells, further supporting the direct role of mitotic cyclins in spindle elongation, but the CDK substrates involved in this process remain unknown [21]. If mitotic CDK promote spindle elongation, cells overexpressing these cyclins are expected to show premature spindle elongation, but these cells exhibit relatively normal spindle elongation, although defect in mitotic exit was noticed [22], [23]. The presence of the CDK inhibitory kinase Swe1 may prevent hyper-activation of mitotic CDK when *CLB2* is overexpressed, as Swe1 specifically inhibits mitotic CDK [24]–[27].

Here we show evidence indicating that overexpression of mitotic cyclin *CLB2* induces premature spindle elongation in *swe1*Δ mutant cells. We further found that FEAR mutants suppress this premature spindle elongation, suggesting that Clb2 induces spindle elongation through FEAR that facilitates Cdc14 release during early anaphase. In contrast to mitotic cyclins, we found that S phase cyclin Clb5 plays a negative role in spindle elongation. Therefore, mitotic CDK likely activates the FEAR pathway to alleviate the inhibitory effect of S-phase CDK on spindle elongation. Our data further suggest that the phosphorylation of a SPB protein Spc110 by S-phase CDK contributes at least partially to the inhibition of spindle elongation. Moreover, high levels of S-phase CDK prevent microtubule localization of Stu2, a microtubule-plus-end tracking protein essential for spindle elongation. Strikingly, overexpression of *CLB2* in securin mutants *pds1*Δ leads to uncoupled sister-chromatid separation and spindle elongation. Given the established role of securin Pds1 in the synchronous chromosome segregation [5], our data support the possibility that this securin-dependent synchrony and the temporal control of spindle elongation by the balance of mitotic versus S-phase CDK ensure the sequential order of sister-chromatid separation and spindle elongation, which is critical for faithful chromosome segregation.

## Results

### Overexpression of mitotic cyclin Clb2 results in premature spindle elongation

**Figure 1:**
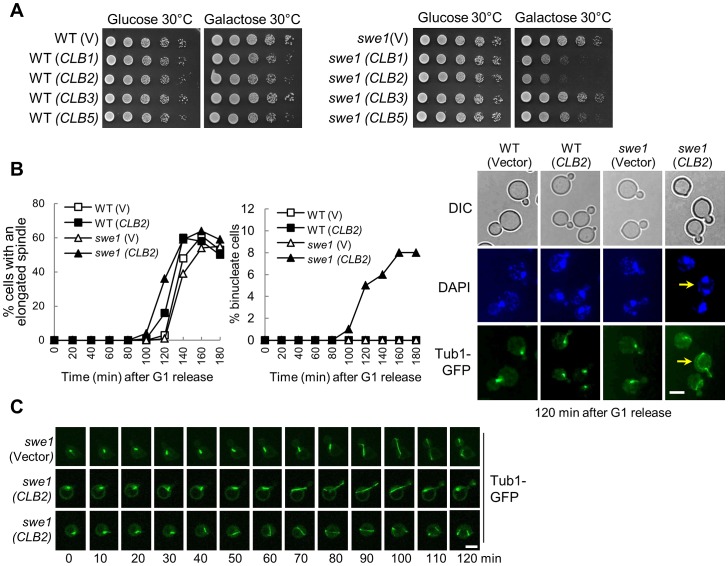
Overexpression of mitotic cyclin *CLB2* results in premature spindle elongation in *swe1Δ* mutants. A. Overexpression of *CLB2* is toxic to *swe1Δ* mutants. WT and *swe1Δ* mutant cells with a control vector or *P_GAL_CLB1*, *P_GAL_CLB2*, *P_GAL_CLB3*, and *P_GAL_CLB5* plasmids were grown to saturation in glucose medium, 10-fold diluted, and spotted onto glucose or galactose plates. The plates were scanned after incubation at 30°C for 3 days. B. Overexpression of *CLB2* in *swe1*Δ mutant cells leads to premature spindle elongation. G_1_-arrested *PDS1-Myc TUB1-GFP* and *swe1Δ PDS1-Myc TUB1-GFP* cells with a vector or a *P_GAL_CLB2* plasmid were released into 30°C galactose medium to induce *CLB2* overexpression. Cells were collected over time and fixed to examine GFP signal and for DAPI staining. Spindles longer than 3 µm were counted as elongated. The percentage of cells with an elongated spindle is shown in the left panel (n>100). The percentage of binucleate cells is shown in the middle panel and the spindle morphology in cells at 120 min time point is shown in the right panel. The arrow indicates a binucleate cell with premature spindle elongation. Scale bar, 5 µm. The budding index, FACS analysis and Pds1 protein level are shown in Figure S2. C. Live-cell imaging shows the premature spindle elongation in *swe1*Δ mutants overexpressing *CLB2*. *swe1*Δ *TUB1-GFP* cells with a vector or a *P_GAL_CLB2* plasmid were arrested in G_1_-phase in raffinose medium. After released into galactose medium for 1.5 hr, the cells were transferred to an agarose pad on a microscope slide to perform live-cell imaging at 25°C. Scale bar, 5 µm.

Clb2 is the major mitotic cyclin in budding yeast, but its overexpression from a galactose-inducible promoter does not cause obvious premature mitosis in wild-type (WT) cells. Because Swe1 kinase phosphorylates and inhibits mitotic CDK, it is possible that the presence of Swe1 prevents the hyper-activation of mitotic CDK after *CLB2* overexpression. Therefore, we overexpressed *CLB2* from a galactose-inducible promoter in *swe1*Δ mutant cells and examined the cell growth. The Western blotting result confirmed the high level expression of Clb2 protein after galactose induction (Figure S1A). Compared to the control cells, *swe1*Δ mutants with a *P_GAL_CLB2* plasmid showed obvious growth defect on galactose plates. Overexpression of Clb1, which is closely related to Clb2, also caused sick growth phenotype in *swe1*Δ cells, but overexpression of Clb3, Clb4, or S phase cyclin Clb5, Clb6 was not toxic (Figure 1A and S1B). Therefore overexpression of mitotic cyclins Clb1 and Clb2 is toxic to *swe1*Δ cells. To confirm that mitotic CDK is hyperactive in *swe1*Δ cells after *CLB2* overexpression, we compared the phosphorylation kinetics of Pol12 in synchronized WT and *swe1*Δ cells overexpressing *CLB2*, as Pol12 is a known substrate of mitotic CDK required for DNA replication [24]. Our results showed that *CLB2* overexpression induces premature Pol12 phosphorylation in both WT and *swe1*Δ cells based on the band-shift, and the phosphorylation became more significant in *swe1*Δ cells as indicated by the increased slow migrating band (Figure S1C). Therefore, the absence of Swe1 indeed causes hyper-activation of mitotic CDK after *CLB2* overexpression.

To understand the cause of this sick growth phenotype, G_1_-arrested WT and *swe1*Δ cells with a control vector or a *P_GAL_CLB2* plasmid were released into galactose medium to induce *CLB2* overexpression and we compared the cell cycle progression in these cells. Both WT and *swe1*Δ cells showed almost identical budding index and DNA synthesis kinetics either with or without *CLB2* overexpression (Figure S2A and S2B). These cells also exhibited similar cell cycle regulated fluctuation of Pds1 protein levels (Figure S2C). However, we noticed premature spindle elongation in some small-budded and unbudded *swe1*Δ cells overexpressing *CLB2*, but this phenotype is much less significant in WT cells (Figure 1B). After G_1_ release into galactose medium for 120 min, 14% of WT cells with a *P_GAL_CLB2* plasmid had elongated spindles (>3 µM), while 36% of *swe1Δ* cells showed elongated spindles. Interestingly, about 8% *swe1*Δ cells became binucleate after G_1_ release for 160 min, i.e. two nuclei were observed in a single cell body (the arrow in Figure 1B). Among them, half were small-budded, while the others were unbudded.

Previous data indicate that overexpression of a single copy of a Clb2 destruction box mutant prevents bud formation and results in binucleate cells [28]. Indeed, we found that some cells overexpressing *CLB2* remained unbudded after G_1_ release (Figure S2A). To further define the role of the inhibition of budding and premature spindle elongation in the formation of binucleate cells, we performed live-cell imaging to examine the dynamics of spindle elongation. G_1_-arrested *swe1*Δ cells with a control vector or a *P_GAL_CLB2* plasmid were released into galactose medium. The spindle elongation in *swe1*Δ (*P_GAL_CLB2*) cells initiated ∼20 min earlier compared to the cells with a control vector. Interestingly, we observed premature spindle elongation in both small-budded and unbudded *swe1*Δ cells (Figure 1C), indicating that both budding inhibition and premature spindle elongation may contribute to the formation of binucleate cells. Because we also observed the binucleate phenotype in both unbudded and small budded cells after Clb2 overproduction (Figure 1B), we reason that the inhibition of budding is not essential for the formation of binucleate cells.

If a cell elongates spindle when cohesion is still present, some sister chromatids may remain together after spindle elongation. However, all the *swe1*Δ cells overexpressing *CLB2* with an elongated spindle showed separated sister chromatids (Figure S3A), indicating that spindle elongation did not occur prior to cohesion dissolution. Alternatively, hyperactive mitotic CDK may promote cohesin cleavage. Thus, we examined Scc1 proteins in WT and *swe1*Δ cells with and without *CLB2* overexpression, but all these cells exhibited similar Scc1 cleavage kinetics based on the appearance of the short Scc1 fragments (Figure S3B), arguing against the possibility that Clb2 induces spindle elongation through cohesin cleavage. We speculate that both hyperactive mitotic CDK and cohesion dissolution are essential for spindle elongation. If that is the case, overexpression of *CLB2* may cause more dramatic premature spindle elongation in cohesin mutant cells. We first found that *scc1-73* mutant cells with *P_GAL_CLB2* plasmids grew more slowly on galactose plates at 25°C compared to control cells (Figure S4A). Moreover, after G_1_ release, *CLB2* overexpression caused premature spindle elongation in *scc1-73* cells (Figure S4B), and this phenotype became more pronounced in *swe1*Δ *scc1-73 cells* (Figure S4C). Therefore, hyperactive mitotic CDK cause more dramatic premature spindle elongation in cells with compromised sister chromatid cohesion.

### Mitotic CDK promotes spindle elongation through the FEAR pathway

**Figure 2:**
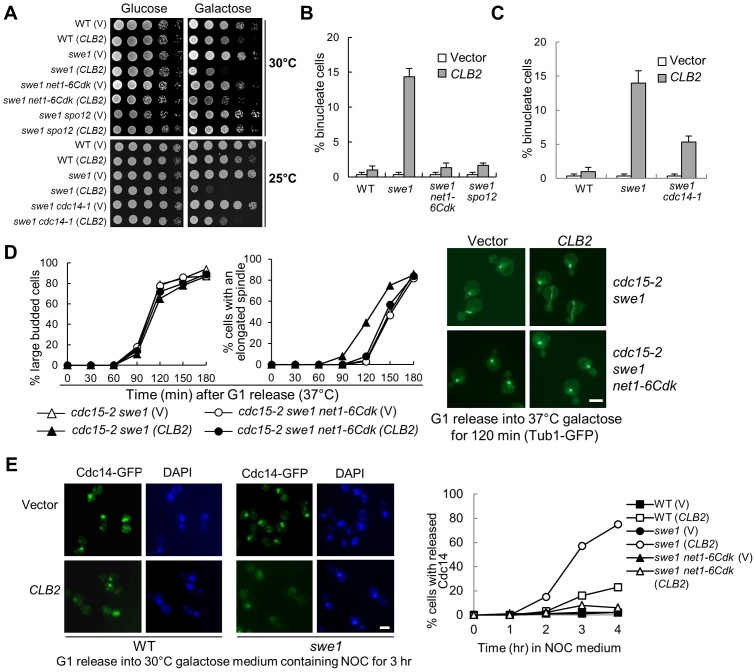
Mitotic CDK promotes spindle elongation through FEAR pathway. A. FEAR and *cdc14-1* mutants suppress the growth defects in *swe1*Δ cells overexpressing *CLB2*. A control vector or a *P_GAL_CLB2* plasmid was transformed into WT, *swe1*Δ, *swe1*Δ *net1-6Cdk*, and *swe1*Δ *spo12*Δ cells. The growth of the transformants on 30°C glucose and galactose plates was examined as described in Figure 1A. The growth of WT, *swe1*Δ, *swe1*Δ *cdc14-1* with a vector control of *P_GAL_CLB2* plasmid on glucose and galactose plates at 25°C is also shown. B. FEAR mutants suppress the binucleate phenotype in *swe1*Δ cells overexpressing *CLB2*. Cells with the indicated genotypes were grown to mid-log phase in raffinose medium and then shifted to galactose medium. The cells were collected after 4 hr incubation at 30°C and fixed for DAPI staining. The percentage of binucleate cells is shown. The experiments were repeated for 3 times and at least 100 cells were counted for each sample. C. *cdc14-1* mutants suppress the binucleate phenotype in *swe1*Δ cells overexpressing *CLB2*. WT, *swe1*Δ and *cdc14-1 swe1*Δ cells with a control vector or a *P_GAL_CLB2* plasmid were grown to mid-log phase in raffinose medium and then switched to galactose medium. After 4 hr incubation at 37°C, the cells were fixed for DAPI staining. The percentage of binucleate cells from three independent experiments is shown (n>100). D. FEAR mutants suppress the premature spindle elongation in *swe1*Δ cells overexpressing *CLB2*. *cdc15-2 swe1*Δ and *cdc15-2 swe1*Δ *net1-6Cdk* cells with a control vector or a *P_GAL_CLB2* plasmid were arrested in G_1_ phase in raffinose medium at 25°C and then released into galactose medium at 37°C. The budding index and the percentage of cells with an elongated spindle are shown in the left panels (n>100). The spindle morphology after 120 min release from G_1_ is shown in the right panel. The scale bar is 5 µm. E. Overexpression of *CLB2* in *swe1*Δ mutant results in premature Cdc14 release. G_1_-arrested *CDC14-5GFP*, *swe1*Δ *CDC14-5GFP* and *swe1*Δ *net1-6Cdk CDC14-5GFP* cells with a vector or a *P_GAL_CLB2* plasmid were released into 30°C galactose medium containing 20 µg/ml nocodazole (NOC). Cells were collected over time and fixed to examine Cdc14 localization. The Cdc14 localization in cells at 3 hr time point is shown in the left panel. The percentage of cells with released Cdc14 was counted over time (right panel, n>100). Scale bar, 5 µm.

One of the substrates of mitotic CDK is the nucleolar Cdc14-binding protein Net1, whose phosphorylation triggers the dissociation of Cdc14 from Net1 and the release of Cdc14 from the nucleolus. It is possible that hyperactive mitotic CDK stimulates spindle elongation by activating FEAR. Because the replacement of 6 CDK phosphorylation sites in Net1 with alanine generates *net1-6Cdk* mutant, which prevents FEAR activation [14], we first compared the growth of *swe1*Δ and *swe1*Δ *net1-6Cdk* cells after *CLB2* overexpression. The *swe1*Δ *net1-6Cdk* cells grew much better than the single mutant cells after *CLB2* overexpression. Another FEAR mutant *spo12*Δ showed an even stronger suppression of the sick growth phenotype of *swe1*Δ (Figure 2A). The nuclear morphology was also compared in *swe1*Δ, *swe1*Δ *spo12*Δ and *swe1*Δ *net1-6Cdk* mutant cells overexpressing *CLB2*, both *spo12*Δ and *net1-6Cdk* mutants suppressed the formation of binucleate cells (Figure 2B), suggesting that the activation of FEAR pathway contributes to the growth defect in *swe1*Δ cells overexpressing *CLB2*. To directly determine if the toxicity of *CLB2* overexpression to *swe1*Δ cells is due to hyperactive Cdc14, we examined the growth of *swe1*Δ *cdc14-1* cells overexpressing *CLB2*. We found that *cdc14-1 swe1*Δ cells with *P_GAL_CLB2* plasmids grew better than *swe1*Δ cells on galactose plates at 25°C (Figure 2A). In addition, the *cdc14-1* mutant partially suppressed binucleate phenotype of *swe1*Δ cells (Figure 2C). We reason that the incomplete suppression is due to the presence of partial functional Cdc14 in *cdc14-1* mutant. Therefore, *CLB2* overexpression likely induces premature spindle elongation by activating Cdc14.

Unlike the FEAR pathway, the mitotic exit network (MEN) induces Cdc14 release in late anaphase [22], [29]. To further test if Clb2-Cdk1 promotes spindle elongation by activating FEAR or MEN, we examined Clb2-induced premature spindle elongation in *cdc15-2 swe1*Δ cells. The abolishment of MEN function in *cdc15-2* mutant did not block Clb2-induced premature spindle elongation. However, the introduction of either *net1-6Cdk* or *spo12*Δ mutation in *swe1*Δ *cdc15-2* cells abolished premature spindle elongation completely (Figure 2D and Figure S5), indicating that Clb2-Cdk1 induces this phenotype through FEAR but not MEN. To directly determine if *CLB2* overexpression in *swe1*Δ cells causes premature FEAR activation, the localization of Cdc14 was examined after G_1_-arrested *swe1*Δ cells were released into galactose medium containing microtubule poison nocodazole that arrests cells in metaphase. Strikingly, 75% of *swe1*Δ cells with a *P_GAL_CLB2* plasmid showed released Cdc14 after incubation in galactose medium for 4 hrs, while only 23% of WT cells showed this phenotype. In contrast, *swe1*Δ cells with a control vector did not show any Cdc14 release. *net1-6Cdk* mutant suppressed the premature Cdc14 release in *swe1*Δ cells (Figure 2E). All these data indicate that excess mitotic CDK likely trigger premature spindle elongation by activating Cdc14 through the FEAR pathway. However, we cannot exclude the possibility that Clb2-Cdk1 also phosphorylates other substrates to promote spindle elongation.

### Excess S-phase CDK delays spindle elongation

**Figure 3:**
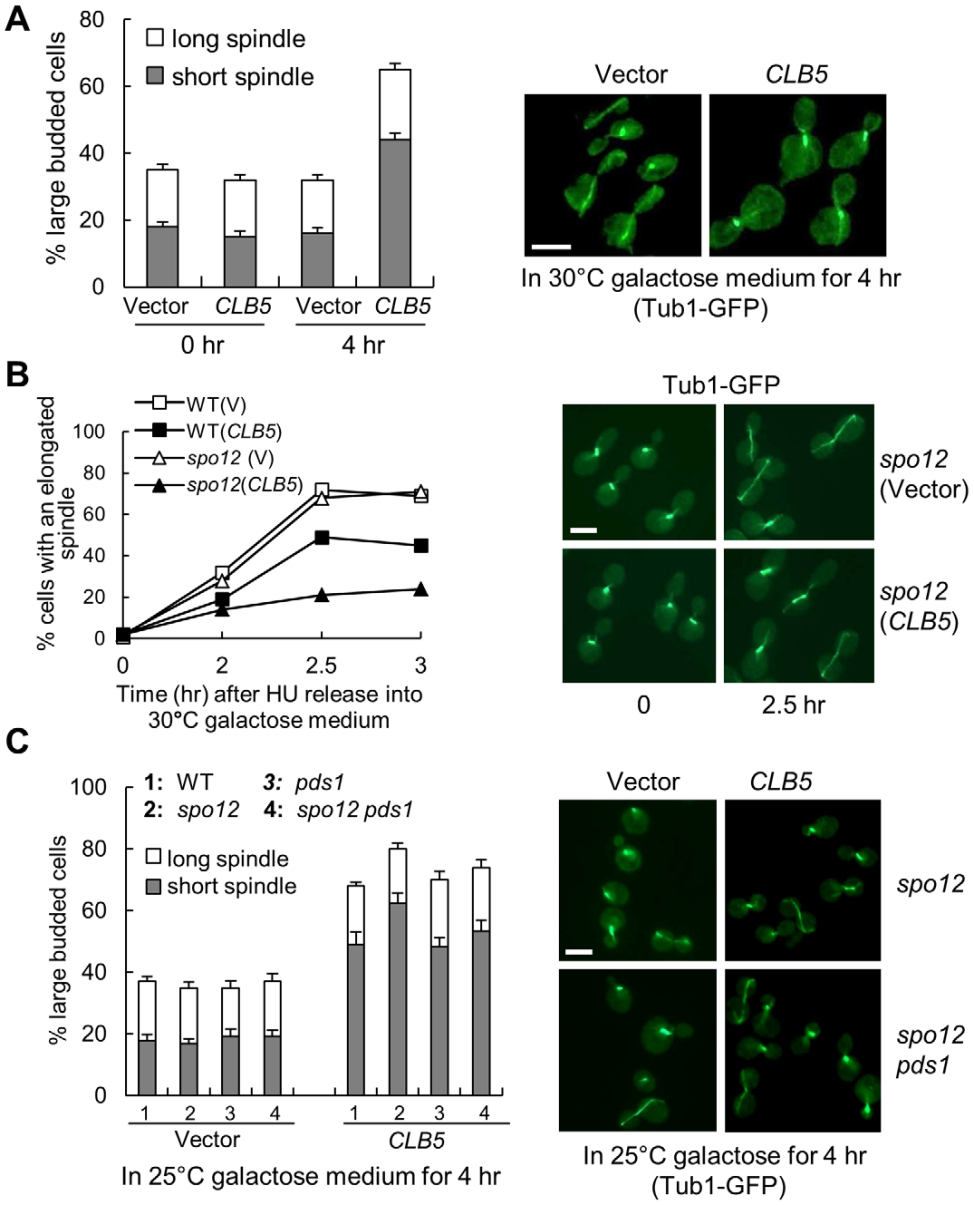
S-phase CDK negatively regulates spindle elongation. A. Overexpression of S-phase cyclin *CLB5* leads to accumulation of cells with a short spindle. *TUB1-GFP* cells with a control vector or a *P_GAL_CLB5* plasmid were grown in raffinose medium to log phase and then shifted into 30°C galactose medium for 4 hrs. The budding index and the percentage of cells with a short (<3 µm) or long spindle are shown in the left panel. The spindle morphology in some representative cells (4 hr in galactose) is shown in the right panel. The experiment was repeated 3 times. The scale bar is 5 µm. B. Overexpression of *CLB5* blocks spindle elongation in cells released from HU arrest. The G_1_-arrested cells with the indicated genotypes were released into 200 mM HU medium and incubated at 30°C for 2 hr. After HU was washed off, the cells were released into 30°C galactose medium and collected over time to examine spindle morphology. The percentage of cells with elongated spindle (>3 µm) is shown in the left panel (n>100). The spindle morphology in some representative cells at time 0 and 2.5 hr is shown in the right panel. Scale bar, 5 µm. C. Deletion of *PDS1* gene does not suppress the spindle elongation defects in cells with high levels of Clb5. *TUB1-GFP* cells with the indicated genotypes were grown in raffinose medium and then shifted to 25°C galactose medium for 4 hr. The experiment was repeated 3 times and the percentage of large budded cells with a short or long spindle is shown in the left panel. The spindle morphology in some representative cells is shown in the right panel. Scale bar, 5 µm.

Overexpression of *CLB5* slows cell growth, suggesting that hyperactive Clb5-Cdk1 may have a negative effect on the cell cycle (Figure 1A). To test if hyperactive S-phase CDK inhibits spindle elongation, we examined the spindle structure in WT cells overexpressing *CLB5*. After incubation in galactose medium for 4 hrs, more yeast cells with a *P_GAL_CLB5* plasmid arrested with a large bud and a very short spindle structure (Figure 3A). Because these spindles are very short, one explanation is that the failure of SPB separation contributes to the spindle elongation defect. Therefore, we examined the spindle elongation kinetics in cells overexpressing *CLB5* after release from hydroxyurea (HU) arrest. HU blocks DNA synthesis and HU-arrested cells have a short spindle with separated SPBs [30]. After HU wash off, *CLB5* overexpression also caused a clear spindle elongation delay as indicated by the accumulation of cells with a bar-like short spindle structure (Figure 3B). This result suggests that the short spindle observed in cells with hyperactive S-phase CDK is not due to SPB separation defect. As we have shown that *CLB5* overexpression blocks nuclear division in FEAR mutants, such as *slk19*Δ and *spo12*Δ [19], we further examined the spindle elongation kinetics in *spo12*Δ mutants with and without *CLB5* overexpression after HU release. Obviously, spindle elongation was largely blocked by *CLB5* overexpression in *spo12*Δ mutant cells (Figure 3B).

The spindle elongation defect could be due to the activation of the DNA damage or the spindle checkpoint that prevents anaphase onset. Because the activation of these checkpoints depends on the stabilization of securin Pds1 [31], [32], deletion of *PDS1* will abolish these checkpoints. We therefore compared the spindle elongation kinetics in WT, *pds1*Δ, *spo12*Δ, and *spo12*Δ *pds1*Δ mutant cells when *CLB5* is overexpressed. Like WT and *spo12*Δ mutants, *pds1*Δ and *spo12*Δ *pds1*Δ mutants also exhibited accumulation of large-budded cells with a short spindle structure after *CLB5* overexpression (Figure 3C), indicating that hyperactive S-phase CDK prevents spindle elongation in a checkpoint-independent manner. Another possibility is that high levels of S-phase CDK block the cleavage of sister chromatid cohesin to prevent spindle elongation. Strikingly, cells overexpressing *CLB5* showed almost identical kinetics for cohesin cleavage compared to control cells (Figure S6A). Moreover, overexpression of *CLB5* also caused delayed spindle elongation in cohesin mutants incubated at the non-permissive temperature (Figure S6B). Therefore, we conclude that S-phase CDK negatively regulates spindle elongation, and this function is unlikely due to the inability of cohesion resolution.

### The absence of S-phase cyclins leads to premature spindle elongation

If S-phase CDK plays a negative role in spindle elongation, we expect that the loss of S-phase cyclins will cause premature spindle elongation. However, both *clb5Δ* and *clb5*Δ *clb6*Δ mutants showed normal spindle elongation kinetics. It is possible that other redundant mechanism, such as the presence of sister-chromatid cohesion, prevents premature spindle elongation in these mutant cells. To test this possibility, we need to examine the spindle elongation kinetics in *clb5*Δ *clb6*Δ mutants in the absence of cohesion. Because the spindle is unstable in cohesin mutant cells and deletion of the spindle checkpoint, such as *MAD2*, suppresses the spindle instability [33], we determined the spindle elongation kinetics in *scc1-73 mad2*Δ and *clb5*Δ *clb6*Δ *scc1-73 mad2*Δ mutants. Consistent with previous data, *scc1-73 mad2*Δ cells elongated spindle with kinetics similar to WT cells, but *clb5*Δ *clb6*Δ *scc1-73 mad2*Δ mutant cells showed premature spindle elongation (Figure S7A). Therefore, the absence of S-phase cyclins leads to premature spindle elongation in the absence of cohesion.

Our data indicate that S-phase and mitotic CDK may play opposing roles in spindle elongation. If that is true, we expect *CLB2* overexpression to cause premature spindle elongation in the absence of S-phase cyclins. Therefore, we examined spindle elongation in *clb5*Δ *clb6*Δ mutant cells with a *P_GAL_CLB2* plasmid and control cells. As expected, *clb5*Δ *clb6*Δ cells showed premature spindle elongation when *CLB2* is overexpressed and these cells grew slowly on galactose medium (Figure S7B). Together, these data support the conclusion that S-phase CDK plays a negative role in spindle elongation, while mitotic CDK plays a positive role in this process.

### The phosphorylation of Spc110 by S-phase CDK inhibits spindle elongation

**Figure 4:**
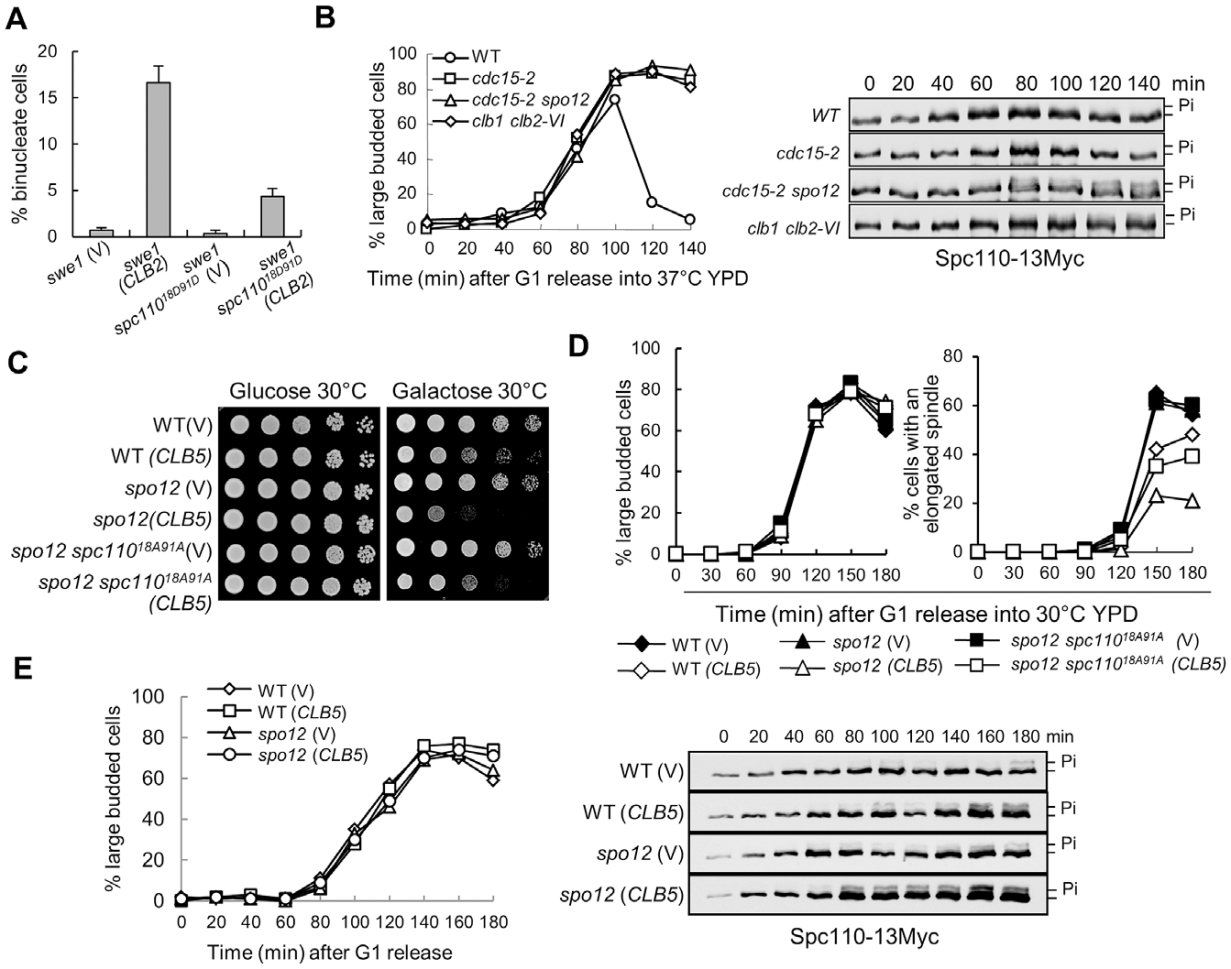
The dephosphorylation of Spc110 promotes spindle elongation. A. *spc110^18D91D^* mutants suppress the binucleate phenotype in *swe1*Δ cells after *CLB2* overexpression. *swe1*Δ and *swe1*Δ *spc110^18D91D^* cells with a control vector or a *P_GAL_CLB2* plasmid were grown to mid-log phase in raffinose medium and then switched to galactose medium. After 4 hr incubation at 30°C, the cells were fixed for DAPI staining. The percentage of binucleate cells is shown after 3 repeats (n>100). B. The dephosphorylation of Spc110 is FEAR dependent. WT, *cdc15-2*, *cdc15-2 spo12*Δ, and *clb1*Δ *clb2-VI* cells with Spc110-13myc were arrested in G_1_ phase at 25°C and then released into cell cycle at 37°C. The protein samples were prepared every 20 min and Western blotting was performed to detect Spc110 phosphorylation. The budding index is shown in the left panel; the Spc110 protein levels and band-shift are shown in the right panel. C. *spc110^18A91A^* mutants partially suppress the growth defects of *spo12*Δ mutant cells overexpressing *CLB5*. Saturated cells with the indicated genotypes were 10-fold diluted and the growth on glucose and galactose plates was examined after 3 day incubation at 30°C. D. *spc110^18A91A^* mutants partially suppress the spindle elongation defects in *spo12*Δ mutants overexpressing *CLB5*. Cells with the indicated phenotypes were arrested in G_1_ phase in raffinose medium and then released into 30°C galactose medium. The percentages of large budded cells and cells with an elongated spindle are shown (n>100). E. Overexpression of *CLB5* in *spo12*Δ mutant cells leads to more dramatic Spc110 phosphorylation. *SPC110-13Myc* and *spo12*Δ *SPC110-13Myc* cells with a vector or a *P_GAL_CLB5* plasmid were arrested in G_1_ phase and released into 30°C galactose medium. The protein samples were prepared every 20 min and Western blotting was performed to detect Spc110 phosphorylation. The budding index is shown in the left panel; the Spc110 protein levels and band-shift are shown in the right panel.

Functional FEAR is required for Clb2-induced premature spindle elongation, and the FEAR promotes Cdc14 release to dephosphorylate Clb5 substrates, such as Ase1 and Fin1 [7], [34], but we found that *ase1*Δ or *fin1*Δ mutant did not suppress Clb2-induced premature spindle elongation. A previous study suggests that Clb5-Cdk1-induced phosphorylation of Spc110, one of the SPB proteins, also modulates spindle dynamics [9]. Interestingly, a phospho-mimetic *spc110^18D91D^* mutant, in which the CDK phosphorylation sites at Thr18 and Ser91 were mutated to aspartic acid, showed dramatic suppression of the binucleate phenotype in *swe1*Δ cells overexpressing *CLB2* (Figure 4A), indicating that the dephosphorylation of Spc110 might be required for Clb2-induced premature spindle elongation. We further compared the spindle elongation kinetics in WT and *spc110^18D91D^* mutant cells and found that the mutant cells did exhibit delayed spindle elongation, although the delay was not pronounced (Figure S8). Nevertheless, the *spc110^18D91D^* mutant failed to rescue the sick growth phenotype of *swe1*Δ cells with *P_GAL_CLB2* on galactose medium, suggesting that phosphorylation of other Clb5 substrates can prevent spindle elongation as well. Alternatively, other unidentified defects induced by Clb2 overexpression may also lead to the sick growth.

The phosphorylation of Spc110 by Clb5-Cdk1 produces a band-shift on protein gels detectable by Western blotting [9]. We have demonstrated that the dephosphorylation of some Clb5-Cdk1 substrates depends on functional FEAR [19]. To test if the FEAR pathway is also required for the dephosphorylation of Spc110, we compared the band-shift of Spc110 protein in WT and mutant cells lacking functional MEN or FEAR. Significant Spc110 phosphorylation was not observed in synchronous WT and MEN mutant cells *cdc15-2*, but we noticed more obvious Spc110 phosphorylation in *cdc15-2 spo12*Δ mutant wherein the function of both MEN and FEAR is abolished, indicating that functional FEAR may be required for Spc110 dephosphorylation. Because mitotic CDK activates the FEAR by phosphorylating Net1, we also examined Spc110 phosphorylation in *clb1*Δ *clb2-VI* temperature sensitive mutants. These mutant cells exhibited more Spc110 phosphorylation, which supports the notion that mitotic CDK promotes Spc110 dephosphorylation (Figure 4B).

We previously showed that overexpression of S-phase cyclin *CLB5* is toxic to FEAR mutants and delays nuclear separation in the mutant cells [19]. If Clb5-induced phosphorylation of Spc110 contributes to this phenotype, a nonphosphorylatable *spc110* mutant will suppress this phenotype. We first found that a nonphosphorylatable *spc110^18A91A^* mutant partially restored the growth of FEAR mutants (*spo12*Δ) with a *P_GAL_CLB5* plasmid on galactose medium (Figure 4C). The spindle elongation dynamics was also examined in *spo12*Δ and *spo12*Δ *spc110^18A91A^* mutants overexpressing *CLB5*. Clb5 overproduction in *spo12*Δ mutants significantly delayed spindle elongation. After G_1_ release for 150 min, 45% of WT cells exhibited elongated spindles, while only 23% of s*po12*Δ mutant cells did. However, 35% of *spo12Δ spc110^18A91A^* mutant cells displayed elongated spindle at 150 min (Figure 4D), indicating that active S-phase CDK prevents spindle elongation at least partially through Spc110 phosphorylation. Consistently, more significant Spc110 phosphorylation was observed in *spo12Δ* cells overexpressing *CLB5* (Figure 4E). In these cells, the kinetics of DNA synthesis is indistinguishable with or without *CLB5* overexpression based on the FACS analysis. Collectively, these data reveal the possibility of a negative role of Clb5-dependent Spc110 phosphorylation in spindle elongation.

### Overexpression of *CLB5* decreases the microtubule localization of Stu2

**Figure 5:**
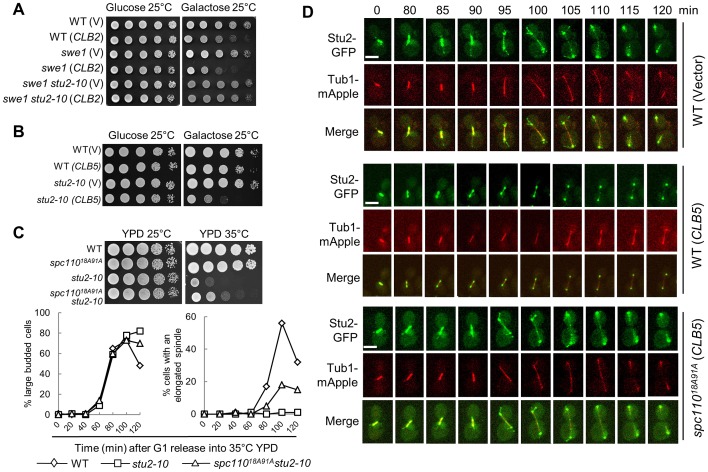
Overexpression of *CLB5* impairs the localization of Stu2 on spindle and cytoplasmic microtubules. A. *stu2-10* temperature-sensitive mutants suppress the growth defect of *swe1*Δ cells overexpressing *CLB2*. The cell cultures with indicated genotypes were 10-fold diluted and then spotted onto glucose and galactose plates. The growth was examined after 4 day incubation at 25°C. B. Overexpression of *CLB5* is toxic to *stu2-10* mutants. The growth of WT and *stu2-10* mutant cells with a control vector or *P_GAL_CLB5* plasmids at 25°C were examined as described in A. C. *spc110^18A91A^* mutants partially suppress the temperature sensitivity and spindle elongation defects of *stu2-10* mutants. The growth of cells with the indicated genotypes at 25°C and 35°C is shown in the top panel. The cells with indicated genotypes were arrested at G_1_ phase at 25°C and then released into YPD medium at 35°C. The budding index and the percentage of cells with an elongated spindle (>3 µm) are shown in the bottom panel (n>100). D. Overexpression of *CLB5* impairs the localization of Stu2 on spindle and cytoplasmic microtubules. WT and *spc110^18A91A^* cells with *STU2-GFP TUB1-mApple* harboring a control vector or a *P_GAL_CLB5* plasmid were arrested with 200 mM HU in raffinose medium for 2.5 hrs. After the cells were released into galactose medium for 1 hr, they were subjected to live-cell microscopy at 25°C. The Stu2 localization and spindle morphology are shown (a reprehensive from more than 10 cells). The scale bar is 5 µm.

Our data suggest that the phosphorylation of SPB component Spc110 plays a role in the timing control of spindle elongation, and this phosphorylation is regulated by the balance of S-phase and mitotic CDKs. As a SPB component, however, it is likely that the phosphorylation of Spc110 regulates the spindle elongation via other microtubule-associated protein(s). Stu2 is the yeast homologue of the XMAP215 protein that binds to the microtubule plus-end [35], [36]. This protein is a processive microtubule polymerase essential for spindle elongation [37], [38]. One possibility is that the CDK activity controls the timing of spindle elongation by regulating the activity of Stu2. Interestingly, the temperature sensitive mutant *stu2-10* dramatically suppressed the toxicity of *CLB2* overexpression to *swe1*Δ mutant cells when incubated at 25°C (Figure 5A), indicating that intact Stu2 function is required for *CLB2*-induced premature spindle elongation. In contrast, *stu2-10* mutant cells were more sensitive to *CLB5* overexpression than WT cells (Figure 5B), indicating that Clb5 may negatively regulates Stu2 function. As we have showed that the phosphorylation of Spc110 by Clb5-Cdk1 plays a negative role in spindle elongation (Figure 4C and 4D), we further compared the growth and spindle elongation in *stu2-10* and *stu2-10 spc110^18A91A^* at 35°C. The results showed that nonphosphorylatable *spc110* mutant partially suppressed the temperature sensitivity and the spindle elongation defect of *stu2-10* mutant cells (Figure 5C), suggesting that S-phase CDK-dependent Spc110 phosphorylation may down-regulate Stu2 function.

To further test if the spindle localization of Stu2 is impaired in cells overexpressing *CLB5*, live-cell imaging was performed in cells with and without *CLB5* overexpression. Consistent with previous reports, we found that Stu2 localized on both spindle poles and spindle at metaphase and early anaphase [36]. At some cell cycle stages, the localization of Stu2 on the cytoplasmic microtubules was also clearly observed in the control cells (Figure 5D). Although the SPB-localization of Stu2 remained similar, we noticed that overexpression of *CLB5* significantly decreased the Stu2 localization on spindle and cytoplasmic microtubules. However, *spc110^18A91A^* mutant restored the microtubule-localization of Stu2 in cells overexpressing *CLB5* (Figure 5D), which is consistent with the result that *spc110^18A91A^* partially suppressed the temperature sensitivity of *stu2-10*. Therefore, we speculate that the phosphorylation of Spc110 by S-phase CDK prevents the localization of Stu2 on spindle and cytoplasmic microtubules, presumably at the plus-ends. In contrast, Spc110 dephosphorylation likely facilitates the microtubule localization of Stu2, which promotes spindle elongation.

### Overexpression of mitotic cyclin *CLB2* in *pds1*Δ mutants results in uncoupled sister-chromatid separation and spindle elongation

Our data indicate that the balance of mitotic and S-phase CDKs regulates the timing of spindle elongation. In addition, the presence of sister chromatid cohesion prevents spindle elongation. Although cohesion mutant cells (*scc1-73*) exhibit premature spindle elongation when *CLB2* is overexpressed, most of the cells have a successful mitosis because they are viable after *CLB2* overexpression. We suspect that an additional mechanism also plays a role in the coordination of cohesion cleavage and spindle elongation.

**Figure 6:**
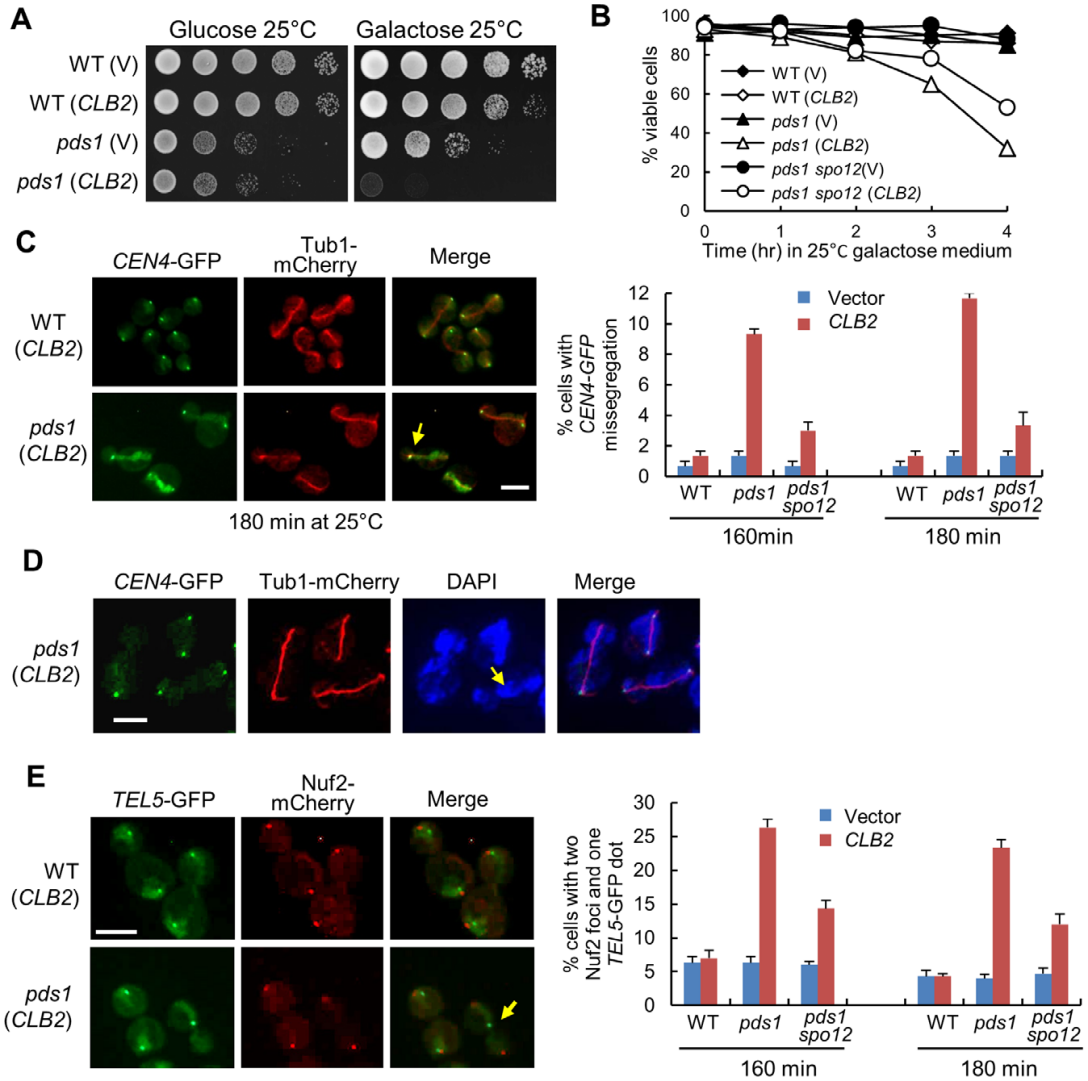
Overexpression of *CLB2* in *pds1*Δ mutants causes uncoupled sister-chromatid separation and spindle elongation. A. Saturated cells with the indicated genotypes were examined for the growth on glucose and galactose plates after 4 day incubation at 25°C. B. Cells with the indicated genotypes were grown to mid-log phase in raffinose medium and then shifted to galactose medium. Cells were collected over time and the viability was examined using platting efficiency (n>300). C. WT, *pds1*Δ, and *pds1*Δ *spo12*Δ cells in *CEN4*-*GFP TUB1-mCherry* background with a vector or a *P_GAL_CLB2* plasmid were arrested in G_1_-phase and then released into 25°C galactose medium to induce *CLB2* overexpression. Cells were collected at 160 and 180 min and fixed to examine chromosome segregation. The spindle morphology and *CEN4-*GFP segregation in some representative cells at 180 min are shown in the left panel. The arrow indicates a cell with an elongated spindle and mis-segregated *CEN4-*GFP. The average percentage of cells with mis-segregated *CEN4-*GFP at 160 and 180 min from three experiments is shown in the right panel (n>100). Scale bar, 5 µm. D. WT and *pds1*Δ cells in *CEN4*-*GFP TUB1-mCherry* background with a vector or *P_GAL_CLB2* plasmid were grown to mid-log phase in raffinose medium and then shifted to galactose medium. The cells were collected after 4 hr incubation at 25°C and fixed for DAPI staining. The segregation of *CEN4-*GFP as well as the spindle and nuclear morphology in some representative cells is shown. The scale bar is 5 µm. E. WT, *pds1*Δ, and *pds1*Δ *spo12*Δ cells in *TEL5-GFP NUF2-mCherry* background were treated as described in C. The experiment was repeated for 3 times. The segregation of telomere of chromosome V (*TEL5-*GFP) in cells at 180 min is shown in the left panel. The arrow indicates a cell with unseparated *TEL5*. The average percentage of cells with two Nuf2 foci and one *TEL5-*GFP dot at 160 and 180 min is shown in the right panel after three repeats (n>100). Scale bar, 5 µm.

Securin Pds1 binds to and inhibits separase Esp1, whose activity is required for the cleavage of cohesion Scc1/Mcd1 and the subsequent anaphase onset [2]. Thus, cell cycle-regulated Pds1 protein levels control the timing of cohesion cleavage and anaphase onset. A previous study showed that deletion of a nonessential gene *CDH1* caused lethality in *pds1*Δ cells and expression of an extra copy of *SWE1* suppressed this lethality [39]. Because *CDH1* encodes an APC activator required for Clb2 degradation, it is possible that the high Clb2 levels contribute to the synthetic lethality of *cdh1 pds1* mutants. Therefore, we examined the growth of *pds1*Δ cells overexpressing *CLB2* and found that overexpression of *CLB2* was very toxic to *pds1*Δ cells (Figure 6A). One possibility is that high levels of mitotic CDK cause chromosome biorientation defects [40], which require the intact spindle assembly checkpoint for survival. However, overexpression of *CLB2* was not toxic to checkpoint mutant *mad2*Δ (Figure S9), excluding the possibility that the checkpoint defect in *pds1*Δ contributes to the lethality. After *CLB2* expression for 4 hrs, 68% of *pds1*Δ cells lost viability (Figure 6B). A FEAR mutant *spo12*Δ failed to suppress the growth defect of *pds1*Δ (*P_GAL_CLB2*) cells on galactose plates, but it partially suppressed the lethality of *pds1*Δ (*P_GAL_CLB2*) cells after incubation in liquid galactose media (Figure 6B). Therefore, we conclude that high level of Clb2 proteins causes the lethality in *pds1*Δ mutant cells.

The presence of Pds1 prevents the activation of separase Esp1 and the abrupt Pds1 degradation prior to anaphase onset allows a robust cohesion cleavage, resulting in synchronous dissolution of all chromosome pairs. The absence of Pds1 decreases this synchrony [5], thus, the loss of this synchrony may cause catastrophic mitosis when *CLB2* is overexpressed. To test this possibility, we first examined the spindle elongation and sister centromere separation in *pds1*Δ cells overexpressing *CLB2*. After *pds1*Δ cells were released from G_1_-arrest into galactose medium for 3 hrs, about 12% cells exhibited an elongated spindle with a single *CEN4-*GFP dot, indicating the failure of chromosome IV separation after spindle elongation (Figure 6C). Given that this number is only for one of the 16 chromosomes, the defect in chromosome segregation should be very dramatic and quick viability loss supports this speculation. We found that the mis-segregation of *CEN4-*GFP was largely suppressed by *spo12*Δ, which is consistent with the notion that *CLB2* promotes spindle elongation through FEAR (Figure 6C).

We further used DAPI staining to examine the chromosome segregation in *pds1*Δ cells overexpressing *CLB2*. Strikingly, most of the cells failed to show two clear DNA masses after spindle elongation. Instead, they exhibited lagged DNA along the elongated spindle (Figure 6D), indicating a remarkable chromosome segregation defect. The observation of cells with an elongated spindle and a single *CEN4*-GFP dot supports this speculation. We further examined the segregation of the telomere of chromosome V (*TEL5-*GFP) in *pds1*Δ cells overexpressing *CLB2*. After G_1_ release into galactose for 180 min, 23% of *pds1*Δ cells exhibited a single *TEL5-*GFP dot with two kinetochore clusters (Nuf2-mCherry) separated to two daughter cells, indicating the failure of telomere separation for chromosome V (Figure 6E). *spo12*Δ also showed partial suppression of *TEL5-*GFP mis-segregation (Figure 6E). Interestingly, the percentage of *pds1*Δ cells with a single *TEL5-*GFP dot after *CLB2* overexpression is obviously higher than that with unseparated *CEN4-*GFP (23% vs. 11%). Our explanation is that both unseparated and partially separated chromosomes contribute to the telomere separation defect. The results support the possibility that overexpression of *CLB2* in *pds1*Δ cells induces spindle elongation when cohesin still holds a few chromosomes or some parts of a chromosome. Nevertheless the small amount of cohesin is unable to restrain the premature spindle elongation induced by *CLB2* overexpression, thereby resulting in the failure for the segregation of entire or part of a chromosome. Premature spindle elongation in *pds1*Δ mutant cells, therefore, induces uncoupled chromosome segregation and spindle elongation, which leads to significant chromosome mis-segregation.

## Discussion

**Figure 7:**
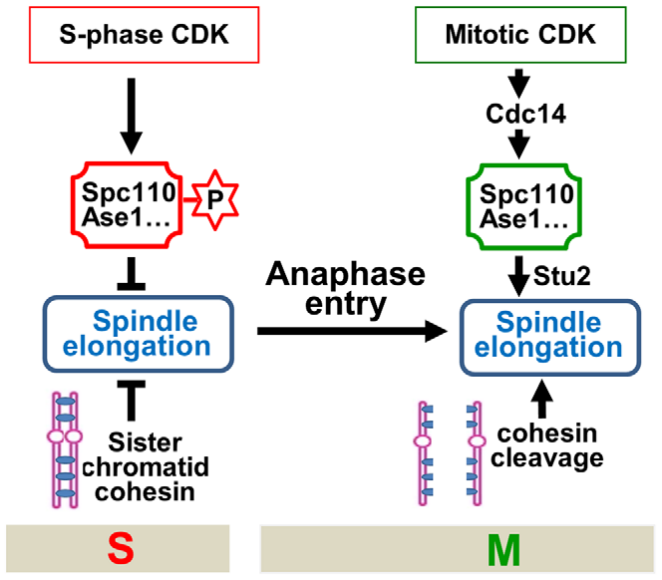
The working model for the timing control of spindle elongation by the balance of mitotic versus S-phase CDKs.

The key to a successful cell division is the coordination of various cell cycle events. For efficient chromosome segregation, spindle elongation should follow the dissolution of sister-chromatid cohesion in an orderly fashion. The molecular mechanism that ensures this sequential order remains unclear. The absence of premature spindle elongation in cells lacking cohesion indicates a cohesion-independent mechanism that controls the timing of spindle elongation. Here we show that S-phase CDK negatively regulates spindle elongation, while mitotic CDK actives the FEAR pathway to trigger Cdc14 release, which reverses S-phase CDK-dependent protein phosphorylation and simulates spindle elongation. Therefore, the balance of mitotic vs. S-phase CDK activity is critical for the timing control of spindle elongation. We also show that S-phase CDK prevents spindle elongation in part by phosphorylating a SPB component Spc110, while dephosphorylation of Spc110 by Cdc14 likely facilitates the localization of Stu2, a plus-end tracking protein, to spindle microtubules, which may directly promotes spindle elongation by enhance microtubule polymerization [37]. Furthermore, hyperactive mitotic CDK in *pds1Δ* cells, where the synchrony of chromosome segregation is compromised, leads to uncoupled sister-chromatid separation and spindle elongation, resulting in chromosome mis-segregation and cell death. A model illustrating and integrating this cell cycle regulatory network is shown in Figure 7.

These findings reveal at least two mechanisms that prevent premature spindle elongation. Firstly, before anaphase onset, S-phase CDK phosphorylates several microtubule-associated proteins to prevent spindle elongation. Therefore, in addition to promoting DNA synthesis, S-phase CDK also negatively regulates mitosis to ensure the correct order of S and M phase spindle function [41]. Secondly, sister-chromatid cohesion restrains spindle elongation as well. To allow spindle elongation, both the loss of sister-chromatid cohesion and the reversion of protein phosphorylation imposed by S-phase CDK have to be achieved. Before anaphase onset, the destruction of Pds1 frees separase Esp1 that cleaves cohesin rings. Nevertheless, the reversion of S-phase CDK-dependent phosphorylation requires the destruction of S-phase cyclin Clb5 as well as the activation of phosphatase Cdc14. Clb5 is degraded before anaphase onset along with Pds1 through APC^Cdc20^ [18]. The release of Esp1 from the inhibition by Pds1 not only triggers cohesin cleavage but also activates FEAR to release Cdc14 [15]. Therefore, this mechanism ensures that cohesin cleavage and spindle elongation are coordinated temporally (Figure 7).

The tightly regulation of the protein levels of securin Pds1 during cell cycle not only avoids the dissolution of sister-chromatid cohesion prior to anaphase entry [42], but also ensures that all chromosome pairs disjoin almost simultaneously [5]. Loss of this switch-like mechanism in *pds1Δ* mutant cells results in asynchronous cohesion dissolution. Therefore, in *pds1Δ* mutant cells, it is possible that only a few sister chromatids are linked by cohesin to restrain spindle elongation while cells have initiated spindle elongation. Surprisingly, most *pds1Δ* cells are able to segregate chromosomes successfully. The temporal control mechanism described above may prevent spindle elongation when cohesion is partially dissolved in *pds1Δ* mutants, but deregulation of this temporal control mechanism could lead to catastrophic mitosis. Indeed, we showed that overexpression of *CLB2* in *pds1Δ* mutants results in the failure of the separation of some sister chromatids after spindle elongation. In addition to high levels of mitotic cyclins, constitutive activation of FEAR by deletion of *CDC55* or the deletion of S-phase cyclins has also been shown to be lethal to *pds1Δ* mutants [39], [43], [44], and the induction of premature spindle elongation is likely the cause of the lethality. Because only some sister-chromatids are linked by cohesin rings when spindles elongate in these cells, the pulling force may break the kinetochore-microtubule interaction, resulting in chromosome mis-segregation. It is also possible that cohesin only exists in part of a chromosome, such as the arm or telomere regions while the spindle is elongating. Under this situation, sister centromeres segregate successfully, but the telomeres of this chromosome still stay together.

Previous work validates the role of mitotic CDK in spindle elongation, as cells lacking both Clb1 and Clb2 fail to elongate the spindle. Because overexpression of Cdc14 phosphatase cannot suppress the spindle elongation defect in *clb1Δ clb2-IV* mutant, it was speculated that mitotic CDK promotes spindle elongation in FEAR-independent manner [21]. Our observation that FEAR mutants completely suppress Clb2-induced premature spindle elongation and toxicity to *swe1Δ* cells strongly supports the conclusion that mitotic CDK promotes spindle elongation through the FEAR pathway. However, we cannot exclude the possibility that additional mitotic CDK targets might also be involved in spindle elongation.

An interesting question is whether cohesin cleavage and spindle elongation occur at the same time. We found that spindle elongation happens earlier in *swe1Δ* mutant cells overexpressing *CLB2*, but the cells with elongated spindle always show separated sister chromatids. Moreover, our result indicates that *CLB2* overexpression does not leads to premature cohesin cleavage. These results suggest that spindle elongation may occur later than cohesin cleavage, but hyperactive CDK eliminates this lag. Because FEAR mutants block Clb2-induced premature spindle elongation in *swe1Δ* mutants, we speculate that the activation of FEAR occurs later than cohesin cleavage, which contributes to the time difference. Therefore, an important function of the FEAR pathway is likely to ensure the sequential order of cohesin cleavage and spindle elongation. Although the hyperactive FEAR in *cdc55Δ* mutant cells did not result in dramatic mitotic defect, *cdc55Δ bub2Δ* double mutant cells exhibit catastrophic mitosis, wherein both FEAR and MEN are hyperactive [16].

Several microtubule-associated proteins, such as Ase1, Fin1, and Ask1, are phosphorylated more efficiently by S-phase CDK and their phosphorylation regulates spindle dynamics [8], [34], [45]. Here we show that S-phase CDK-mediated phosphorylation of Spc110 also plays an important role in spindle elongation. We found that the phospho-mimetic *spc110^18D91D^* mutant partially abolishes Clb2-induced premature spindle elongation. In contrast, the nonphosphorylatable *spc110^18A91A^* mutant suppresses the Clb5-induced delay in spindle elongation. Although *spc110^18D91D^* mutant cells show a noticeable delay in spindle elongation, the delay is not significant. We reason that the phosphorylation of a group of proteins by Clb5-Cdk1 prevents spindle elongation, but the phospho-mimetic mutant for each single protein may not be sufficient to block spindle elongation completely. In contrast to Ase1 and Fin1, which directly bind to microtubules and regulate microtubule dynamics, Spc110 is a SPB component, so it is likely that Spc110 phosphorylation regulates spindle elongation in an indirect way. We show that overexpression of *CLB5* clearly impairs the localization of Stu2 on spindle and cytoplasmic microtubules. The dephosphorylation of Spc110 and/or other Clb5 substrates likely promotes the localization of Stu2 to the plus-end of spindle microtubules, where Stu2 triggers the polymerization and spindle elongation. Further studies are needed to understand how S-phase CDK prevents microtubule localization of Stu2.

In summary, our data reveal the molecular mechanism that coordinates chromosome segregation and spindle elongation. Before anaphase, S-phase CDK and sister chromatid cohesion prevent spindle elongation. The establishment of chromosome bipolar attachment triggers the degradation of both securin Pds1 and S-phase cyclin Clb5. The disappearance of Pds1 activates Esp1 that cleaves cohesin and triggers Cdc14 release through FEAR. Consequently, the loss of sister chromatid cohesion and the reversion of S-phase CDK-dependent protein phosphorylation trigger spindle elongation. This mechanism becomes essential in cells with decreased synchrony of chromosome segregation, as induction of premature spindle elongation in these cells results in unseparated or partially separated chromosomes after spindle elongation. Like budding yeast Clb5, the S-phase cyclin (Cyclin A) in mammalian cells also exhibits substrate specificity [46]. Moreover, the proteins involved in this regulation, such as Ase1, Spc110, and Stu2, are well conserved in higher eukaryotic cells. For example, XMAP215, the Stu2 homologue in mammalian cells, is required for microtubule polymerization and spindle elongation [47]. Therefore, the mechanism of the temporal control of spindle elongation described in budding yeast could be conserved. We represent data showing that the temporal control of spindle elongation is critical for accurate chromosome segregation, suggesting that defects in this network may contribute to aneuploidy that is associated with cancer progression.

## Materials and Methods

### Yeast strains and growth

The yeast strains used in this study are listed in Table S1. All strains are isogenic to Y300, a W303 derivative. Yeast cells were grown in YPD (Yeast extract, Peptone, Dextrose) or indicated synthetic medium. To arrest cells in G_1_ phase, 5 µg/ml α-factor was added into cell cultures. After 2 hr incubation, the G_1_-arrested cells were washed twice with water and then released into fresh medium to start the cell cycle. Nocodazole was used at 20 µg/ml in 1% DMSO. To induce cyclin overexpression, galactose was added to the medium to a final concentration of 2%.

### Cytological techniques

Cells with GFP, mCherry or mApple-tagged proteins were fixed with 3.7% formaldehyde for 5 min at room temperature, and then washed twice with 1× PBS buffer and resuspended in PBS buffer for fluorescence microscopy (Carl Zeiss MicroImaging, Inc.). The spindle morphology was monitored by using *TUB1-GFP*, *TUB1-mApple*, or *TUB1-mCherry* strains and we count the spindles longer than 3 µm as elongated spindles. For DAPI staining, cells were fixed with 3.7% formaldehyde for 5 min at room temperature, and then resuspended in 100% MEOH at −20°C for 30 min. The cells were incubated in DAPI solution (final concentration of 2.5 µg/ml) for 1 min at room temperature. For each experiment, we repeated 3 times and at least 100 cells were counted for each sample.

Live-cell microscopy was carried out with a Nikon Eclipse Ti imaging system (Andor). We used a glass depression slide to prepare an agarose pad filled with synthetic complete medium with the addition of galactose. All live-cell images were acquired at 25°C with an ×100 objective lens. Twelve Z-sections were collected at each time point, and each optical section was set at 0.5 µm thick. The time-lapse interval was set at 5 min. Maximum projection from applicable time points were created using Andor IQ2 software.

### FACS analysis

G_1_ phase-arrested cells in raffinose medium were released into galactose medium. Samples were taken at various time points and fixed in 70% EtOH overnight at 4°C. Cells were then incubated in Tris pH 7.8 buffer with 0.2 mg/ml RNase A at 37°C for 4 hrs and stained with 30 µg/ml propidium iodide at 4°C overnight. FACS analysis was performed using FACSCanto equipped with the FACSDiva software.

### Protein techniques

Cell pellets from 1.5 ml of cell culture were resuspended in 200 µl 0.1 N NaOH and incubated at room temperature for 5 min. After centrifugation, the cells were resuspended in equal volume (30 µl) of ddH_2_O and SDS protein-loading buffer. The samples were then boiled for 5 min and resolved with 8% SDS-polyacrylamide gel. Proteins were detected with ECL (Perkin Elmer LAS, Inc.) after probing with anti-Myc or anti-HA antibodies (Covance Research Products, Inc.) and HRP-conjugated secondary antibody (Jackson ImmunoResearch, Inc.).

## Supporting Information

Figure S1 Expression of *CLB2* from a galactose inducible promoter. A. Clb2 levels in cells overexpressing *CLB2*. G_1_-arrested WT cells with a vector or a *P_GAL_CLB2-HA* plasmid in raffinose medium were released into glucose or galactose medium at 30°C. The protein samples were prepared every 30 min and Western blotting was performed to detect Clb2 level. The Pgk1 protein level is shown as a loading control. B. Overexpression of *CLB4* or *CLB6* is not toxic to *swe1*Δ mutants. WT and *swe1*Δ mutant cells with a control vector or *P_GAL_CLB4*, *P_GAL_CLB6* plasmids were grown to saturation in glucose medium, 10-fold diluted, and spotted onto glucose or galactose plates. The plates were scanned after incubation at 30°C for 3 days. C. *CLB2* overexpression leads to enhanced mitotic CDK activity in WT and *swe1*Δ cells. *POL12-Myc* and *swe1*Δ *POL12-Myc* cells with a vector or a *P_GAL_CLB2* plasmid were synchronized in G_1_ phase in raffinose medium and released into cell cycle in galactose medium. The cells were collected over time to detect Pol12 phosphorylation after Western blotting.
(TIF)

Figure S2 The analysis of cell cycle progression in cells overexpressing *CLB2*. A. (The same cells from a single experiment are used for this figure and Figure 1B). WT and *swe1*Δ cells with a control vector or *P_GAL_CLB2* plasmid were arrested in G_1_ phase and then released into 30°C galactose medium. The cells were collected every 20 min for budding index. The percentage of large budded cells is shown in the top and the percentage of unbudded cells is shown in the bottom. B. The cells in panel A were prepared for FACS analysis. C. The cells in panel A were collected over time and Western blotting was performed to detect Pds1 levels. The Pgk1 level is shown as a loading control.
(TIF)

Figure S3 *CLB2* overexpression does not cause premature cohesin cleavage. A. *swe1*Δ cells with a prematurely elongated spindle after *CLB2* overexpression showed separated sister chromatids. G_1_-arrested *CEN4-GFP TUB1-mCherry* and *swe1*Δ *CEN4-GFP TUB1-mCherry* cells with a vector or a *P_GAL_CLB2* plasmid were released into 30°C galactose medium to induce *CLB2* overexpression. The spindle morphology and sister chromatid separation in *swe1*Δ cells at 120 min is shown. Scale bar, 5 µm. B. Cells overexpressing *CLB2* show similar Scc1 cleavage kinetics. G_1_-arrested *SCC1-Myc* and *swe1*Δ *SCC1-Myc* cells with a vector or a *P_GAL_CLB2* plasmid in raffinose medium were released into galactose medium at 30°C. Cells were collected every 15 min for budding index and the preparation of protein samples. Western blotting was performed to detect Scc1 cleavage. The budding index is shown in the left panel. The full-length and cleaved Scc1 protein levels are shown in the right panel. The Pgk1 level is used as a loading control.
(TIF)

Figure S4 Overexpression of *CLB2* in *scc1-73* mutant cells leads to sick growth and premature spindle elongation. A. Saturated cell cultures with the indicated genotypes were 10-fold diluted and spotted on to glucose and galactose plates. The growth was examined after 4 day incubation at 25°C. B. G_1_-arrested *TUB1-GFP* and *scc1-73 TUB1-GFP* cells with a vector or a *P_GAL_CLB2* plasmid were released into 37°C galactose medium to induce *CLB2* overexpression. Cells were collected over time and fixed to examine spindle morphology. The budding index and the percentage of cells with an elongated spindle are shown (n>100). C. Overexpression of *CLB2* in *scc1-73 swe1*Δ double mutant cells leads to more dramatic premature spindle elongation. *TUB1-GFP*, *scc1-73 TUB1-GFP*, *swe1*Δ *TUB1-GFP* and *scc1-73 swe1*Δ *TUB1-GFP* cells with a vector or a *P_GAL_CLB2* plasmid were first arrested in G_1_ phase and then released into 37°C galactose medium. The cells were collected over time and fixed to examine spindle morphology. The percentage of cells with an elongated spindle at 100, 120, and 140 min after *CLB2* overexpression is shown (n>100).
(TIF)

Figure S5 A FEAR mutant *spo12*Δ suppresses premature spindle elongation in *swe1*Δ cells overexpressing *CLB2*. *cdc15-2 swe1*Δ and *cdc15-2 swe1*Δ *spo12*Δ cells with a control vector or a *P_GAL_CLB2* plasmid were arrested in G_1_ phase in raffinose medium at 25°C and then released into galactose medium at 37°C. The spindle elongation dynamics were examined over time. The budding index and the percentage of cells with an elongated spindle are shown (n>100).
(TIF)

Figure S6 *CLB5* overexpression does not causes delayed cohesin cleavage. A. G_1_-arrested *SCC1-Myc* cells with a vector or a *P_GAL_CLB5* plasmid in raffinose medium were released into galactose medium at 30°C. The protein samples were prepared every 15 min and Western blotting was performed to detect Scc1 cleavage. The budding index is shown in the left panel. The levels of full-length and cleaved Scc1 proteins are shown in the right panel. The Pgk1 level is shown as a loading control. B. Cohesin mutant does not rescue the delayed spindle elongation in cells overexpressing *CLB5*. G_1_-arrested WT and *scc1-73* cells with a vector or a *P_GAL_CLB5* plasmid were released into 200 mM HU medium for 2 hr at 25°C. After HU was washed off, the cells were released into 37°C galactose medium and collected over time to examine spindle morphology. The percentage of cells with elongated spindle (>3 µm) is shown (n>100).
(TIF)

Figure S7 The absence of S-phase cyclins leads to premature spindle elongation. A. *clb5*Δ *clb6*Δ mutant cells showed premature spindle elongation in the absence of cohesin. WT, *clb5*Δ *clb6*Δ, *scc1-73 mad2*Δ, and *clb5*Δ *clb6*Δ *scc1-73 mad2*Δ cells with *TUB1-GFP* were arrested in G_1_ phase at 25°C and then released into YPD medium at 37°C. The spindle elongation kinetics was examined over time. The budding index and the percentage of cells with an elongated spindle are shown in the top panel (n>100). The spindle morphology at 80 min is shown in the bottom panel. Scale bar, 5 µm. B. Overexpression of *CLB2* results in premature spindle elongation in *clb5*Δ *clb6*Δ mutant cells. The cells with the indicated genotypes were 10 fold diluted and then spotted onto glucose and galactose plates. The growth was examined after 4 day incubation at 25°C (top panel). G_1_-arrested *TUB1-GFP* and *clb5*Δ *clb6*Δ *TUB1-GFP* cells with a vector or a *P_GAL_CLB2* plasmid were released into 25°C galactose medium to induce *CLB2* overexpression. Cells were collected over time and fixed to examine spindle morphology. The budding index and the percentage of cells with an elongated spindle are shown in the bottom panel (n>100).
(TIF)

Figure S8 *spc110^18D91D^* mutants show delayed spindle elongation. WT, *spc110^18A91A^* and *spc110^18D91D^* cells with *TUB1-GFP* were arrested in G_1_-phase and then released into YPD medium at 25°C. The cells were collected over time to examine spindle morphology. The budding index, the percentage cells with a metaphase or anaphase spindle, and the spindle morphology in some cells at 100 min time point are shown. Scale bar, 5 µm.
(TIF)

Figure S9 Overexpression of *CLB2* is not toxic to *mad2*Δ mutants. Saturated cell cultures with indicated genotypes were 10-fold diluted and spotted onto glucose and galactose plates to examine the growth after 3 day incubation at 30°C.
(TIF)

Table S1 The strain list used in this study.
(DOC)
