# Supplementary figures and images for: Coordination of Chromatid Separation and Spindle Elongation by Antagonistic Activities of Mitotic and S-Phase CDKs
Source: PLoS Genet. 2013 Feb 28;9(2):e1003319. doi: 10.1371/journal.pgen.1003319 (PMC3584997; doi:10.1371/journal.pgen.1003319)

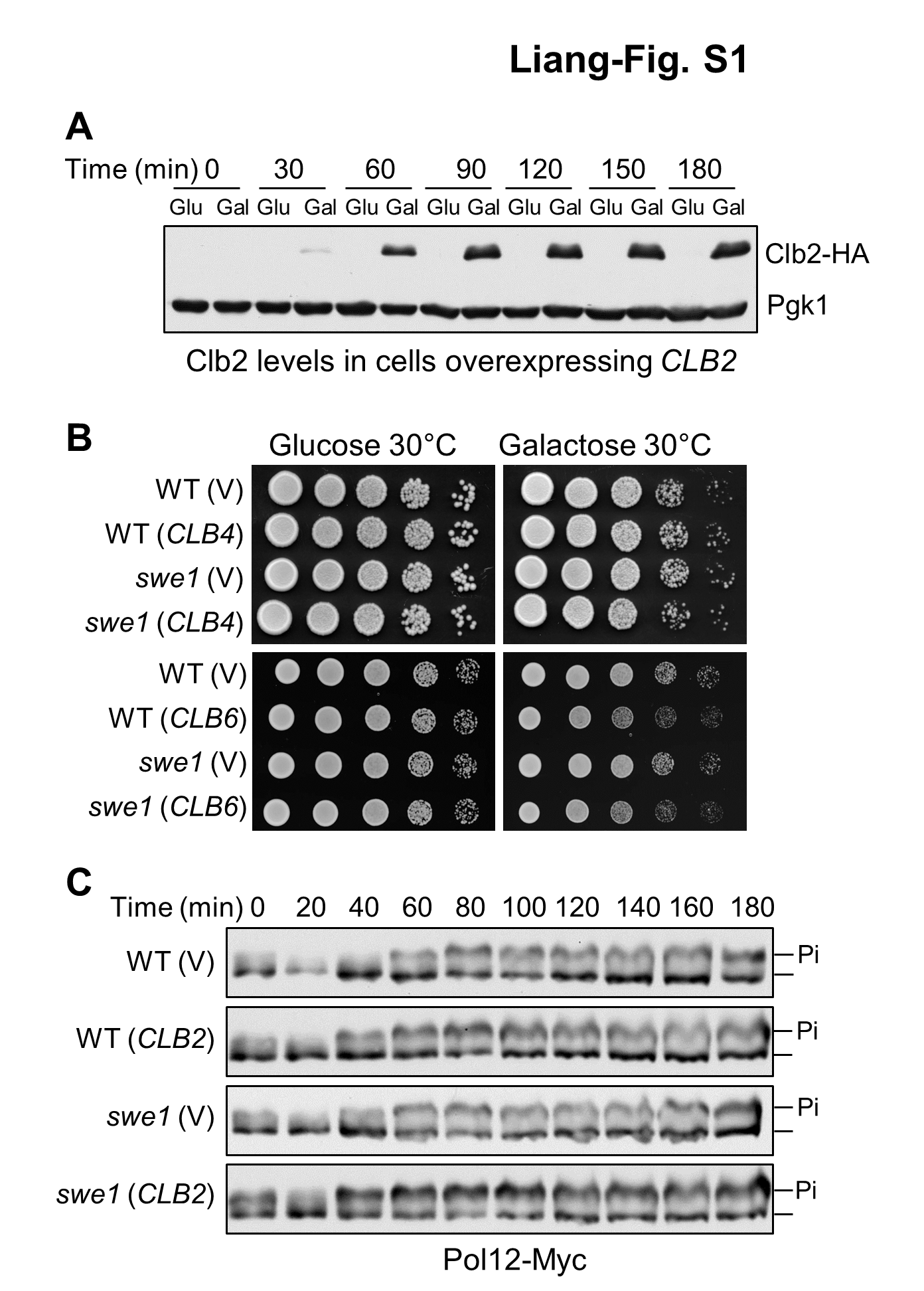

Supplement: Figure S1 — Expression of CLB2 from a galactose inducible promoter. A. Clb2 levels in cells overexpressing CLB2. G1-arrested WT cells with a vector or a PGALCLB2-HA plasmid in raffinose medium were released into glucose or galactose medium at 30°C. The protein samples were prepared every 30 min and Western blotting was performed to detect Clb2 level. The Pgk1 protein level is shown as a loading control. B. Overexpression of CLB4 or CLB6 is not toxic to swe1Δ mutants. WT and swe1Δ mutant cells with a control vector or PGALCLB4, PGALCLB6 plasmids were grown to saturation in glucose medium, 10-fold diluted, and spotted onto glucose or galactose plates. The plates were scanned after incubation at 30°C for 3 days. C. CLB2 overexpression leads to enhanced mitotic CDK activity in WT and swe1Δ cells. POL12-Myc and swe1Δ POL12-Myc cells with a vector or a PGALCLB2 plasmid were synchronized in G1 phase in raffinose medium and released into cell cycle in galactose medium. The cells were collected over time to detect Pol12 phosphorylation after Western blotting. (TIF) [file pgen.1003319.s001.tif]

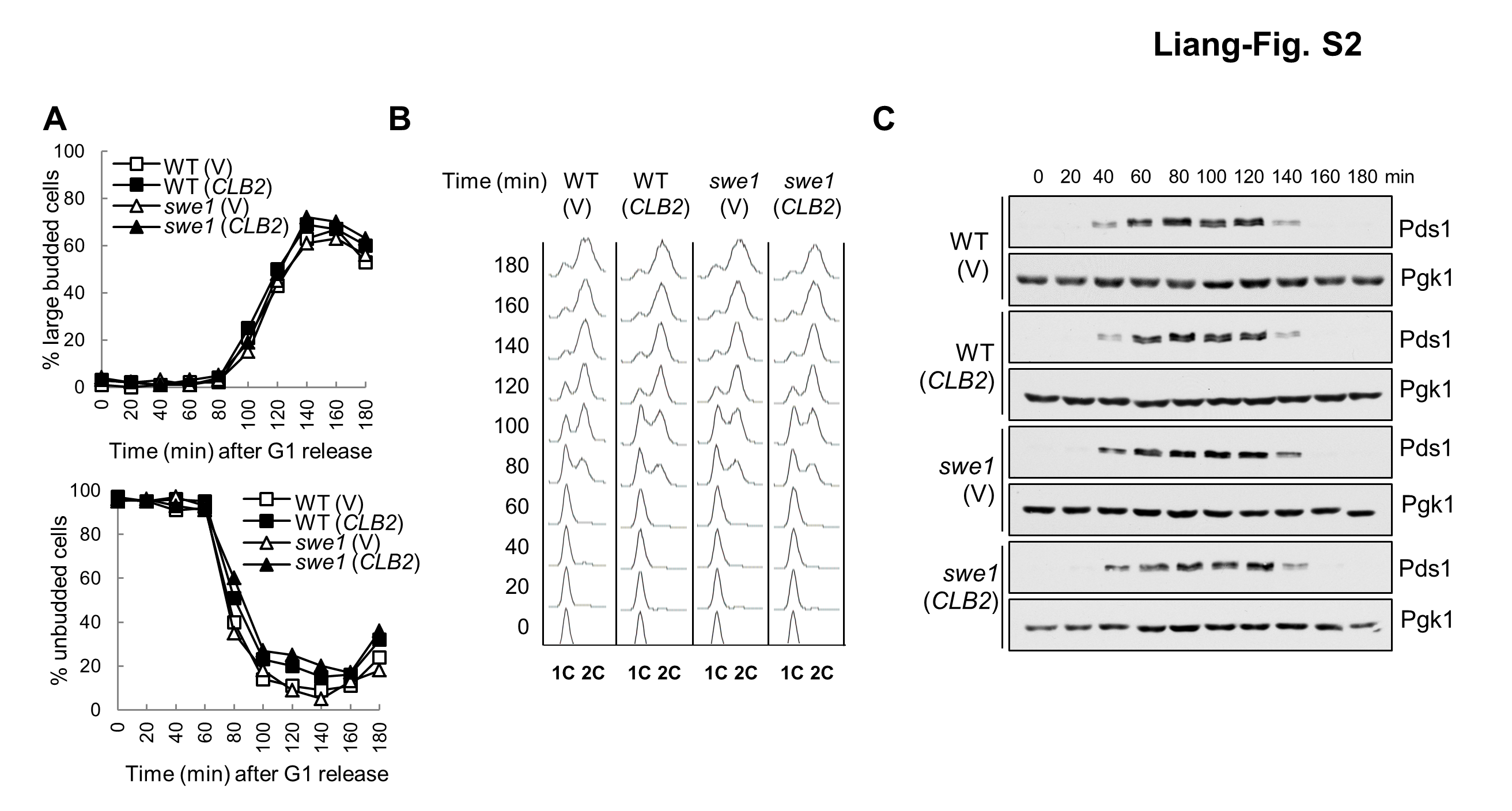

Supplement: Figure S2 — The analysis of cell cycle progression in cells overexpressing CLB2. A. (The same cells from a single experiment are used for this figure and Figure 1B). WT and swe1Δ cells with a control vector or PGALCLB2 plasmid were arrested in G1 phase and then released into 30°C galactose medium. The cells were collected every 20 min for budding index. The percentage of large budded cells is shown in the top and the percentage of unbudded cells is shown in the bottom. B. The cells in panel A were prepared for FACS analysis. C. The cells in panel A were collected over time and Western blotting was performed to detect Pds1 levels. The Pgk1 level is shown as a loading control. (TIF) [file pgen.1003319.s002.tif]

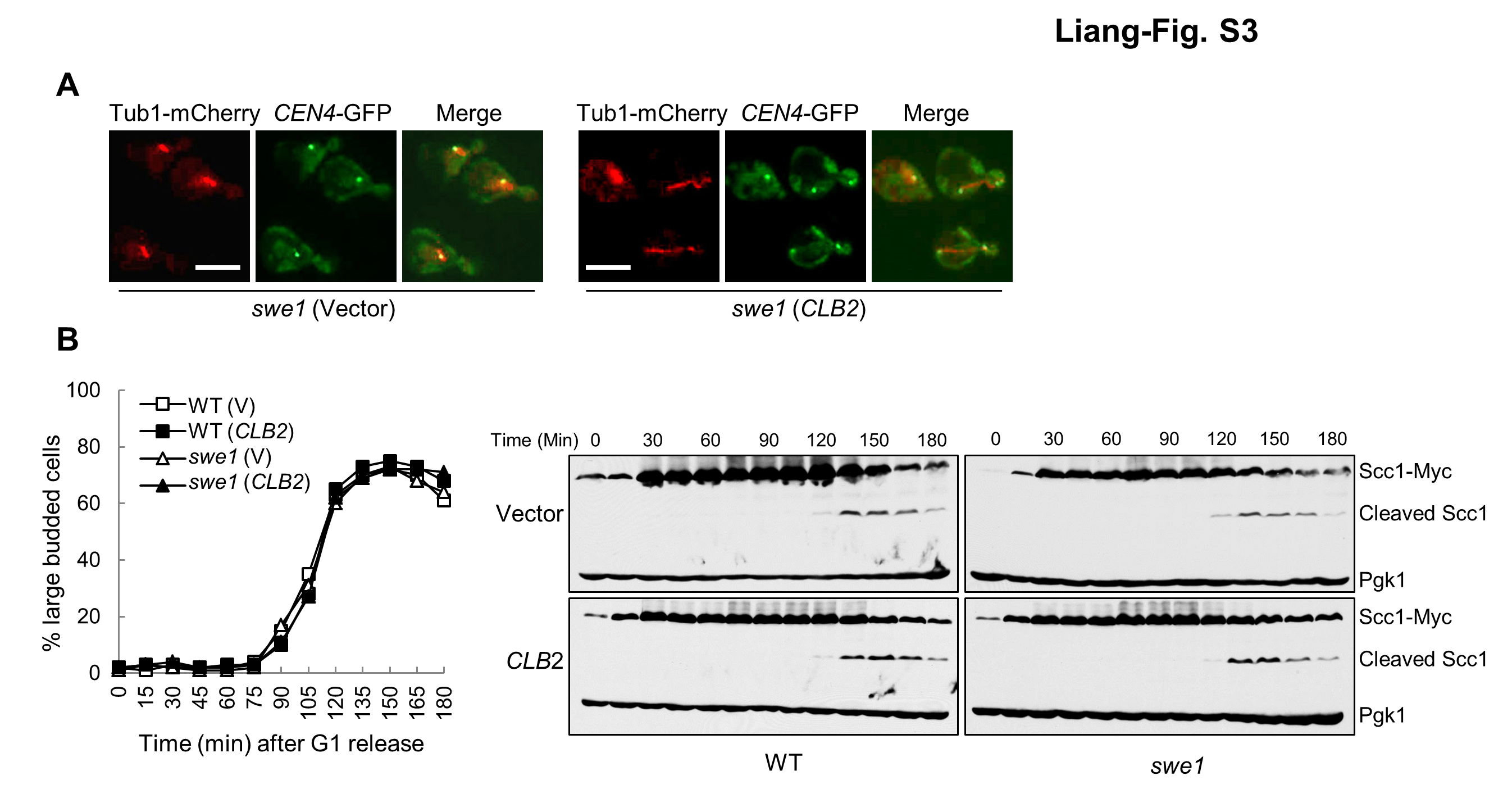

Supplement: Figure S3 — CLB2 overexpression does not cause premature cohesin cleavage. A. swe1Δ cells with a prematurely elongated spindle after CLB2 overexpression showed separated sister chromatids. G1-arrested CEN4-GFP TUB1-mCherry and swe1Δ CEN4-GFP TUB1-mCherry cells with a vector or a PGALCLB2 plasmid were released into 30°C galactose medium to induce CLB2 overexpression. The spindle morphology and sister chromatid separation in swe1Δ cells at 120 min is shown. Scale bar, 5 µm. B. Cells overexpressing CLB2 show similar Scc1 cleavage kinetics. G1-arrested SCC1-Myc and swe1Δ SCC1-Myc cells with a vector or a PGALCLB2 plasmid in raffinose medium were released into galactose medium at 30°C. Cells were collected every 15 min for budding index and the preparation of protein samples. Western blotting was performed to detect Scc1 cleavage. The budding index is shown in the left panel. The full-length and cleaved Scc1 protein levels are shown in the right panel. The Pgk1 level is used as a loading control. (TIF) [file pgen.1003319.s003.tif]

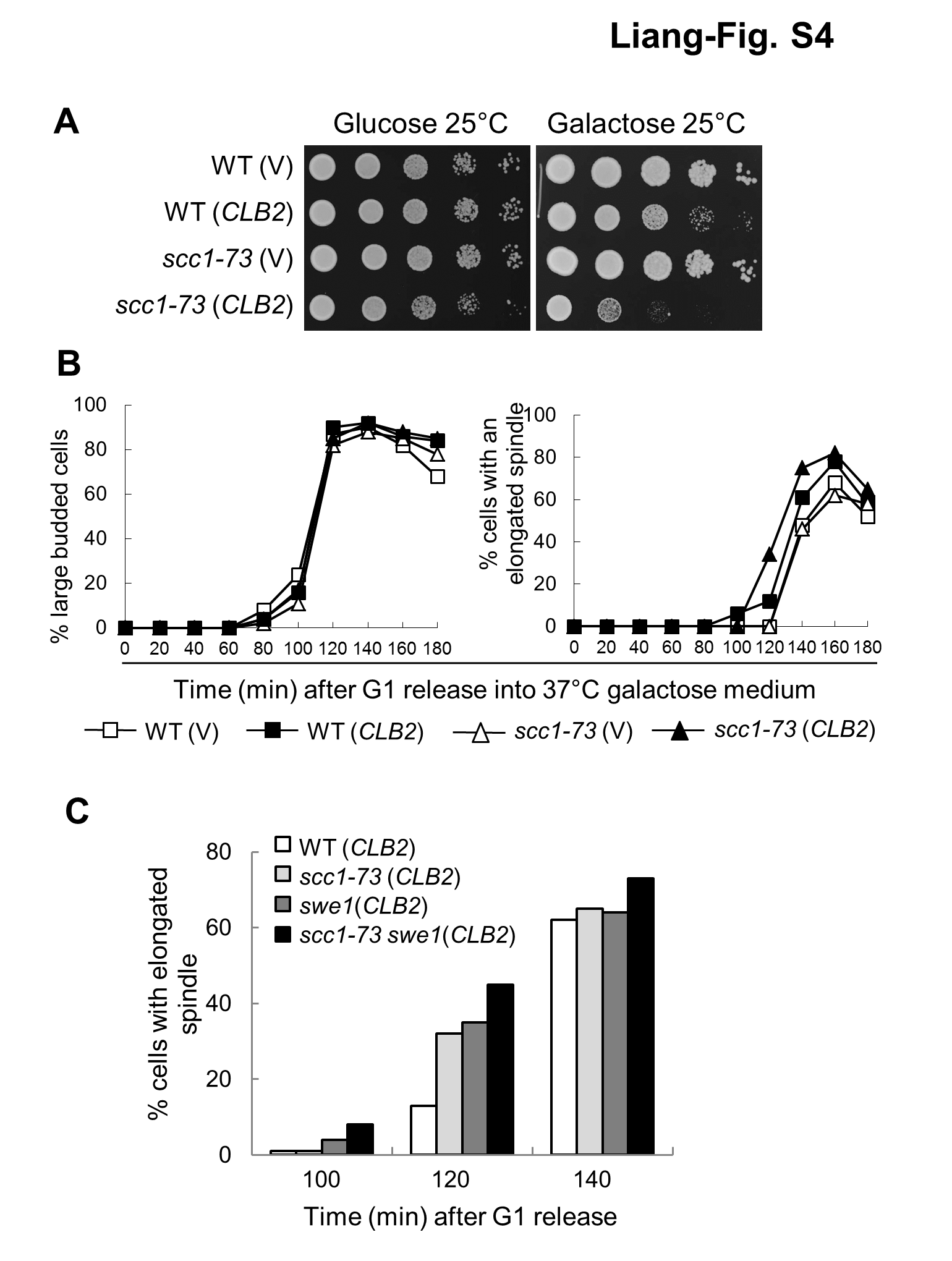

Supplement: Figure S4 — Overexpression of CLB2 in scc1-73 mutant cells leads to sick growth and premature spindle elongation. A. Saturated cell cultures with the indicated genotypes were 10-fold diluted and spotted on to glucose and galactose plates. The growth was examined after 4 day incubation at 25°C. B. G1-arrested TUB1-GFP and scc1-73 TUB1-GFP cells with a vector or a PGALCLB2 plasmid were released into 37°C galactose medium to induce CLB2 overexpression. Cells were collected over time and fixed to examine spindle morphology. The budding index and the percentage of cells with an elongated spindle are shown (n>100). C. Overexpression of CLB2 in scc1-73 swe1Δ double mutant cells leads to more dramatic premature spindle elongation. TUB1-GFP, scc1-73 TUB1-GFP, swe1Δ TUB1-GFP and scc1-73 swe1Δ TUB1-GFP cells with a vector or a PGALCLB2 plasmid were first arrested in G1 phase and then released into 37°C galactose medium. The cells were collected over time and fixed to examine spindle morphology. The percentage of cells with an elongated spindle at 100, 120, and 140 min after CLB2 overexpression is shown (n>100). (TIF) [file pgen.1003319.s004.tif]

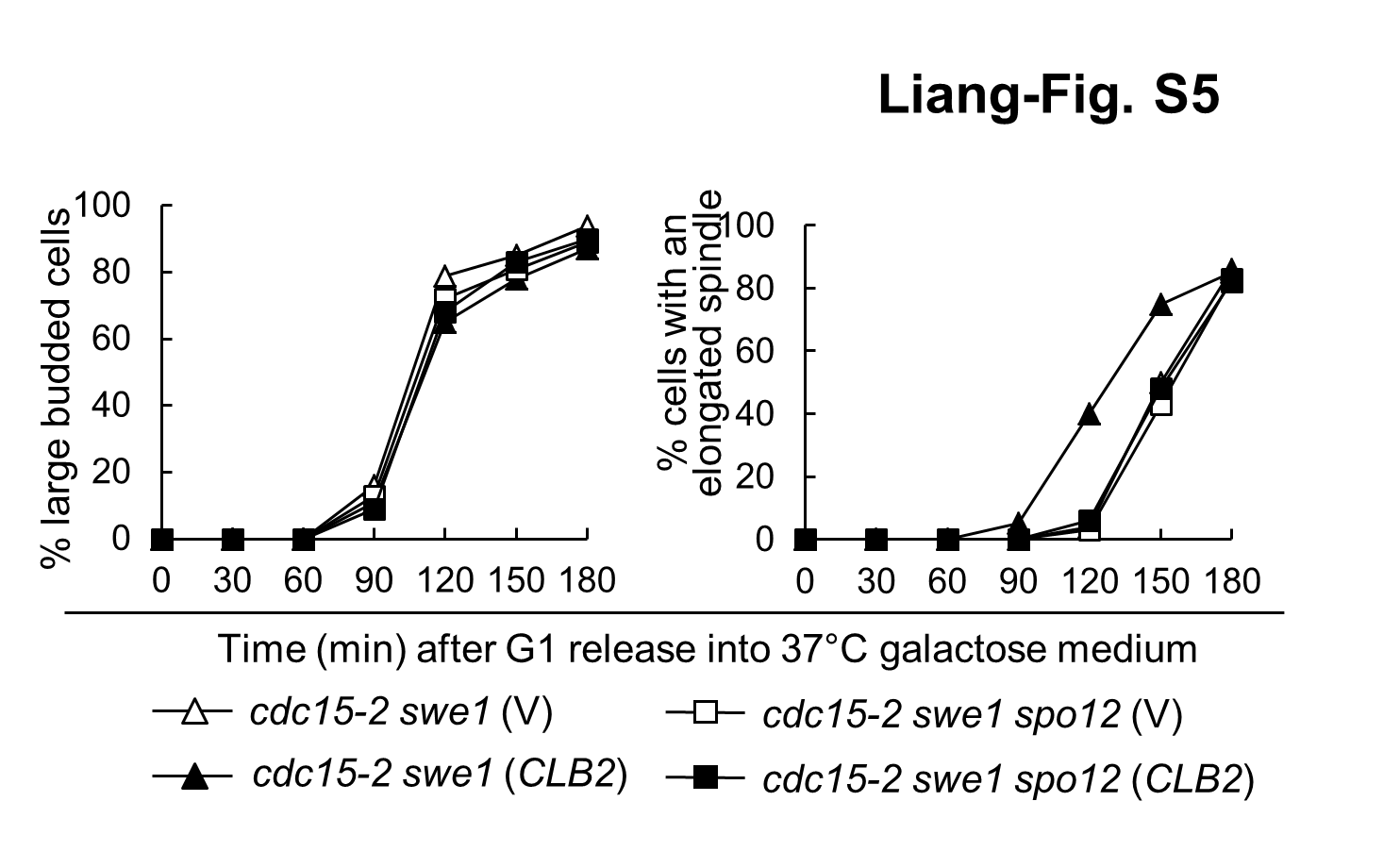

Supplement: Figure S5 — A FEAR mutant spo12Δ suppresses premature spindle elongation in swe1Δ cells overexpressing CLB2. cdc15-2 swe1Δ and cdc15-2 swe1Δ spo12Δ cells with a control vector or a PGALCLB2 plasmid were arrested in G1 phase in raffinose medium at 25°C and then released into galactose medium at 37°C. The spindle elongation dynamics were examined over time. The budding index and the percentage of cells with an elongated spindle are shown (n>100). (TIF) [file pgen.1003319.s005.tif]

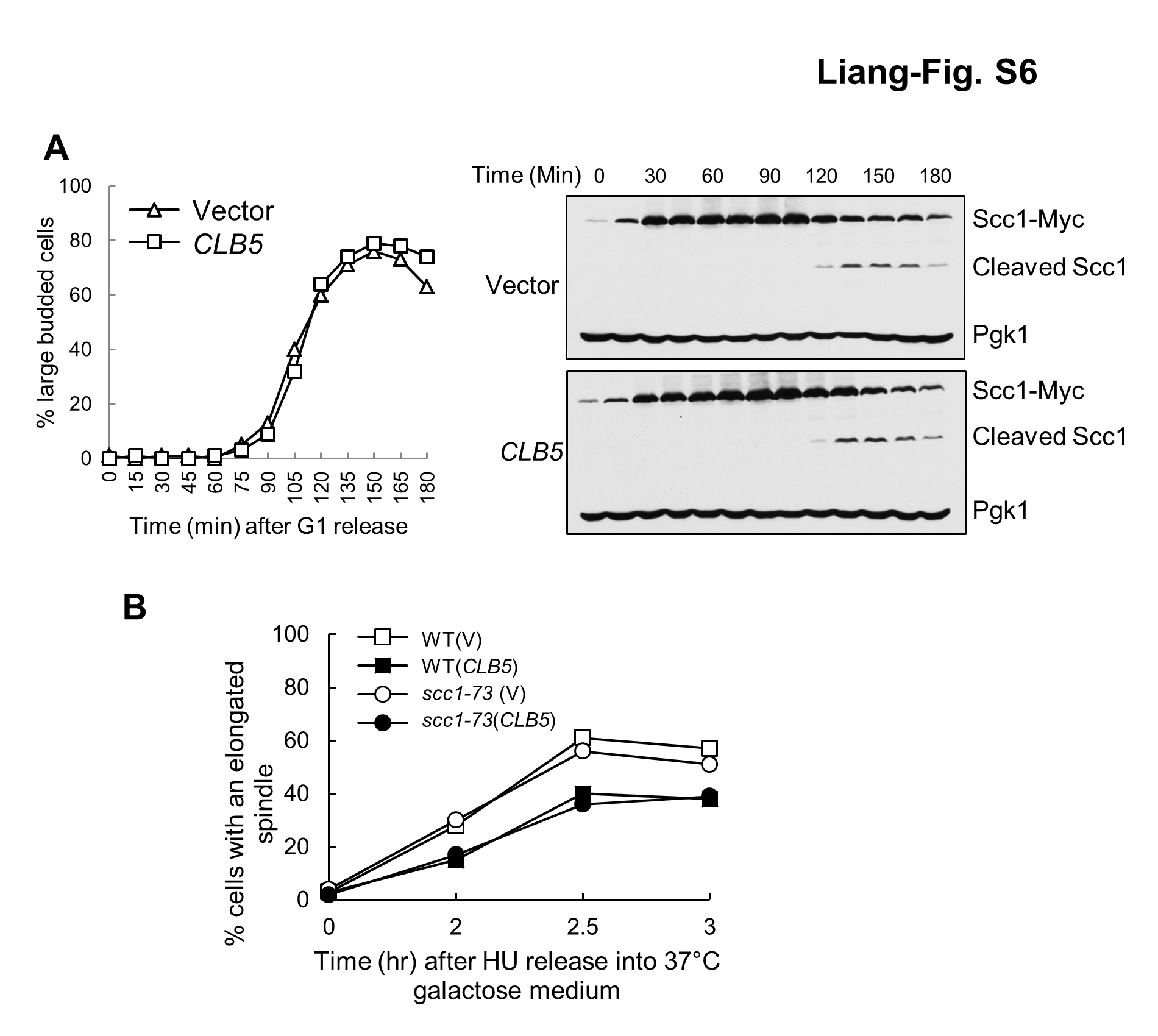

Supplement: Figure S6 — CLB5 overexpression does not causes delayed cohesin cleavage. A. G1-arrested SCC1-Myc cells with a vector or a PGALCLB5 plasmid in raffinose medium were released into galactose medium at 30°C. The protein samples were prepared every 15 min and Western blotting was performed to detect Scc1 cleavage. The budding index is shown in the left panel. The levels of full-length and cleaved Scc1 proteins are shown in the right panel. The Pgk1 level is shown as a loading control. B. Cohesin mutant does not rescue the delayed spindle elongation in cells overexpressing CLB5. G1-arrested WT and scc1-73 cells with a vector or a PGALCLB5 plasmid were released into 200 mM HU medium for 2 hr at 25°C. After HU was washed off, the cells were released into 37°C galactose medium and collected over time to examine spindle morphology. The percentage of cells with elongated spindle (>3 µm) is shown (n>100). (TIF) [file pgen.1003319.s006.tif]

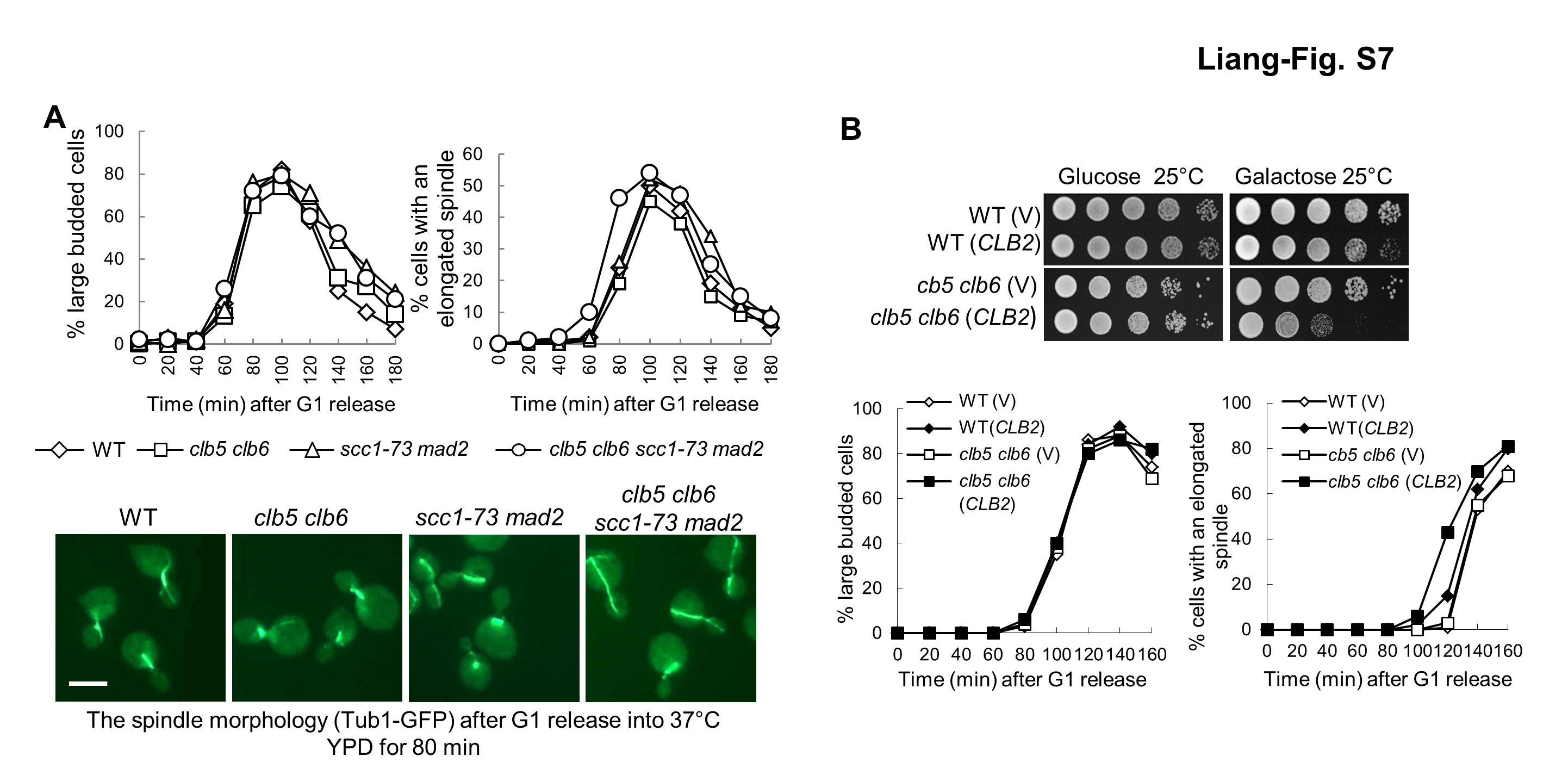

Supplement: Figure S7 — The absence of S-phase cyclins leads to premature spindle elongation. A. clb5Δ clb6Δ mutant cells showed premature spindle elongation in the absence of cohesin. WT, clb5Δ clb6Δ, scc1-73 mad2Δ, and clb5Δ clb6Δ scc1-73 mad2Δ cells with TUB1-GFP were arrested in G1 phase at 25°C and then released into YPD medium at 37°C. The spindle elongation kinetics was examined over time. The budding index and the percentage of cells with an elongated spindle are shown in the top panel (n>100). The spindle morphology at 80 min is shown in the bottom panel. Scale bar, 5 µm. B. Overexpression of CLB2 results in premature spindle elongation in clb5Δ clb6Δ mutant cells. The cells with the indicated genotypes were 10 fold diluted and then spotted onto glucose and galactose plates. The growth was examined after 4 day incubation at 25°C (top panel). G1-arrested TUB1-GFP and clb5Δ clb6Δ TUB1-GFP cells with a vector or a PGALCLB2 plasmid were released into 25°C galactose medium to induce CLB2 overexpression. Cells were collected over time and fixed to examine spindle morphology. The budding index and the percentage of cells with an elongated spindle are shown in the bottom panel (n>100). (TIF) [file pgen.1003319.s007.tif]

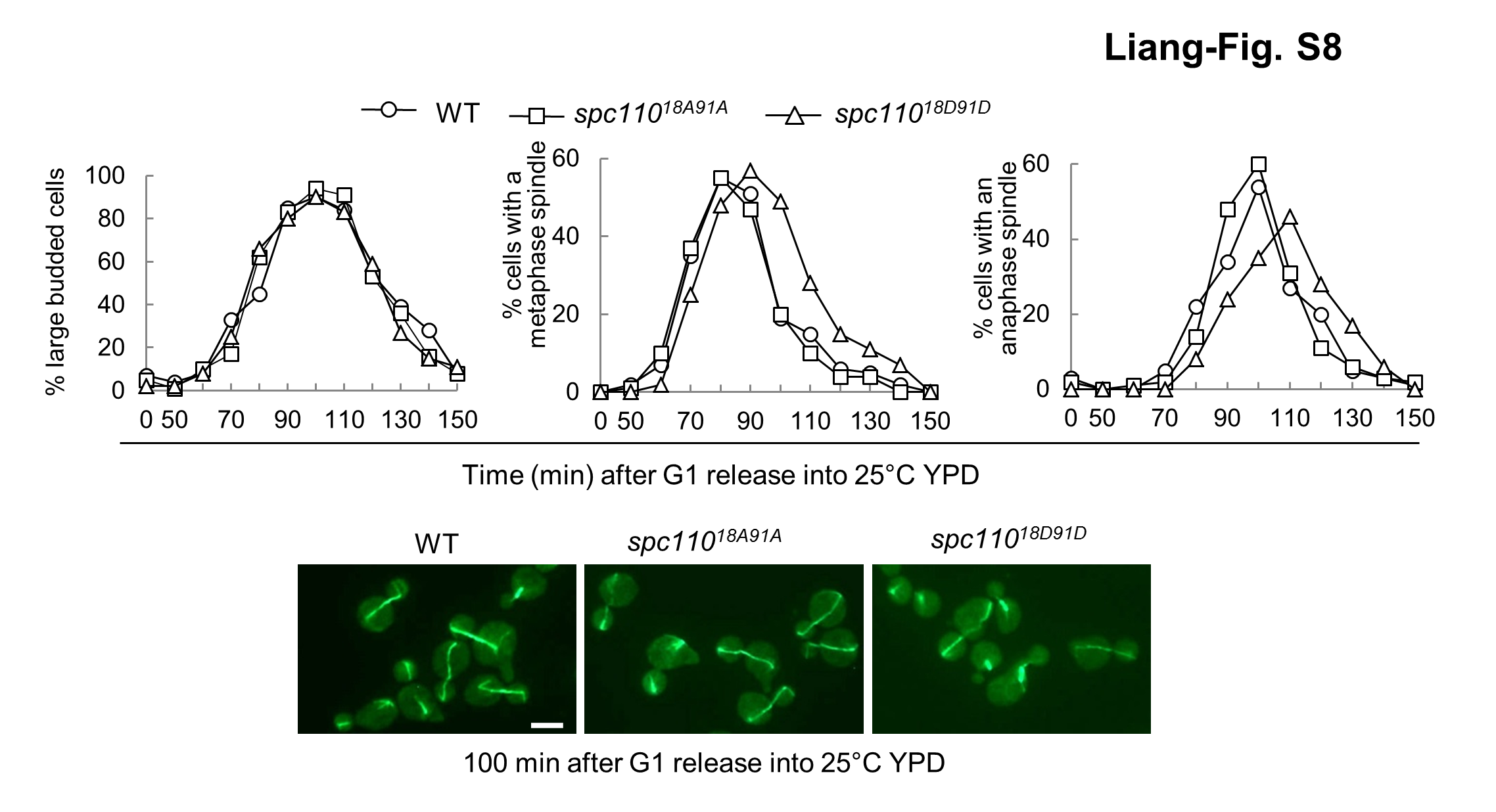

Supplement: Figure S8 — spc11018D91D mutants show delayed spindle elongation. WT, spc11018A91A and spc11018D91D cells with TUB1-GFP were arrested in G1-phase and then released into YPD medium at 25°C. The cells were collected over time to examine spindle morphology. The budding index, the percentage cells with a metaphase or anaphase spindle, and the spindle morphology in some cells at 100 min time point are shown. Scale bar, 5 µm. (TIF) [file pgen.1003319.s008.tif]

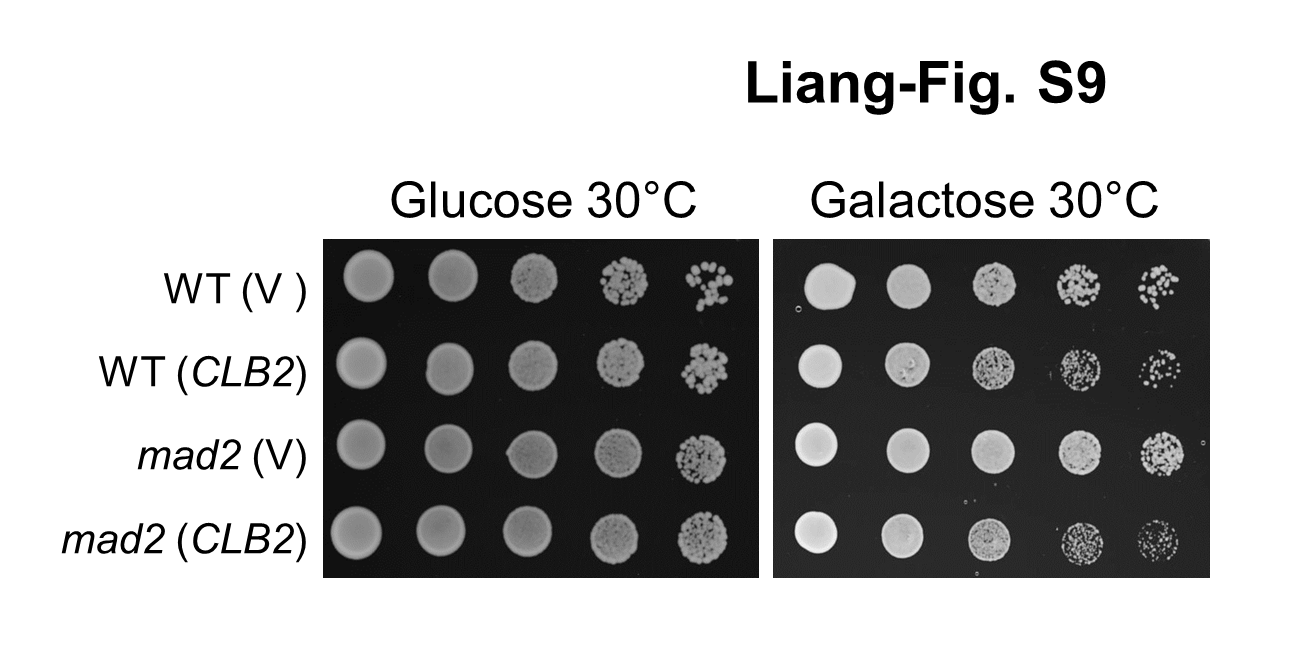

Supplement: Figure S9 — Overexpression of CLB2 is not toxic to mad2Δ mutants. Saturated cell cultures with indicated genotypes were 10-fold diluted and spotted onto glucose and galactose plates to examine the growth after 3 day incubation at 30°C. (TIF) [file pgen.1003319.s009.tif]
