# Supplementary material for: Coordination of Chromatid Separation and Spindle Elongation by Antagonistic Activities of Mitotic and S-Phase CDKs
Source: PLoS Genet. 2013 Feb 28;9(2):e1003319. doi: 10.1371/journal.pgen.1003319 (PMC3584997; doi:10.1371/journal.pgen.1003319)
Supplement: Table S1 — The strain list used in this study. (DOC) [file pgen.1003319.s010.doc]

Table S1: Strains used in this study

| **Stains** | **Related genotypes** | **Source** |
| --- | --- | --- |
| Y300  YYW115  903-1-2  891-7-1  919-7-3  924-1-1  929-2-1  2487-2-1  941-2-2  870-1-2  903-1-1  2560-1-1  2561-16-3  YYW264-1  2216-1-4  2216-9-1  2260-8-4  2274-2-1  SAY801  838-13-2  2520-1-3  2520-5-4  941-23-3  941-2-1  928-20-1  1064-3-3  2007-1-1  2393-3-1  2679-8-4  2167-17-2  2385-1-4  2729-2-3  1080-8-3  2727-7-3  2728-3-3  957-5-4  2868-6-1  1066-4-2  2869-4-1  2803-10-2  JBY705  YYW368  2804-1-3  2863-3-2  2136-1-3  2864-1-1  395-6-3  2866-2-4  2867-1-3  2870-3-3 | *MATa trp1-1 ura3-1 his3-11,15 leu2-3,112 ade2-1 can1-100*  *MATa TUB1-GFP-LEU2*  *MATa swe1Δ::LEU2 TUB1-GFP-LEU2*  *MATa spo12::Kan TUB1-GFP-LEU2*  *MATa swe1Δ::LEU2 net1Δ::Sphis5+ net1-6Cdk-TEV-myc9::TRP1*  *MATa swe1Δ::LEU2 spo12Δ::Kan TUB1-GFP-LEU2*  *MATa pds1Δ::LEU2 TUB1-GFP-LEU2*  *MATa pds1Δ::LEU2 spo12::Kan TUB1-GFP-LEU2*  *MATa clb5Δ::HIS3 clb6Δ::LEU2 TUB1-GFP-LEU2*  *MATa cdc15-2 TUB1-GFP-LEU2*  *MATa cdc15-2 swe1Δ::LEU2 TUB1-GFP-LEU2*  *MATa cdc15-2 swe1Δ::LEU2 spo12Δ::Kan TUB1-GFP-LEU2*  *MATa cdc15-2 swe1Δ::LEU2 net1Δ::Sphis5+ net1-6Cdk-TEV-myc9::TRP1 TUB1-GFP-LEU2*  *MATa SPC110-13myc-Sphis5+*  *MATa cdc15-2 Spc110-13myc-Sphis5+*  *MATa cdc15-2 spo12Δ::Kan SPC110-13myc-Sphis5+*  *MATa clb1Δ::URA3 clb2-VI SPC110-13myc-Sphis5+*  *MATa swe1Δ::LEU2 spc110Δ::Kan spc110^18D91D^-LEU2*  *MATa cdc14::CDC14-5GFP-TRP1* in W303-1A  *MATa swe1Δ::LEU2 cdc14::CDC14-5GFP-TRP1*  *MATa clb5Δ::HIS3 clb6Δ::LEU2 scc1-73 mad2Δ::URA3 TUB1-GFP-LEU2*  *MATa scc1-73 mad2Δ::URA3 TUB1-GFP-LEU2*  *MATa clb5Δ::HIS3 TUB1-GFP-LEU2*  *MATa clb6Δ::LEU2 TUB1-GFP-LEU2*  *MATa scc1-73 TUB1-GFP-LEU2*  *MATa swe1Δ::LEU2 ase1Δ::HIS3*  *MATa swe1Δ::LEU2 fin1Δ::Kan TUB1-GFP-LEU2*  *MATa spc110Δ::Kan spc110^18D91D^-LEU2 TUB1-GFP-URA3*  *MATa spc110Δ::Kan spc110^18A91A^-LEU2 TUB1-GFP-URA3*  *MATa CEN4-GFP TUB1-mCherry*  *MATa pds1Δ::LEU2 CEN4-GFP TUB1-mCherry*  *MATa pds1Δ::LEU2 spo12Δ::Kan CEN4-GFP TUB1-mCherry*  *MATa TEL5-GFP NUF2-mCherry*  *MATa pds1Δ::LEU2 TEL5-GFP NUF2-mCherry*  *MATa pds1Δ::LEU2 spo12Δ::Kan TEL5-GFP NUF2-mCherry*  *MATa swe1Δ::LEU2 CEN4-GFP TUB1-mCherry*  *MATa stu2-10::LEU2 TUB1-GFP-URA3*  *MATa swe1Δ::LEU2 stu2-10::LEU2*  *MATa spc110Δ::Kan spc110^18A91A^-LEU2 stu2-10::LEU2 TUB1-GFP-URA3*  *MATa spc110Δ::Kan spc110^18A91A^-LEU2 spo12Δ::Kan TUB1-GFP-LEU2*  *MATa SCC1-18myc-TRP1*  *MATa STU2-GFP-TRP1 TUB1-mApple-HIS3*  *MATa spc110Δ::Kan spc110^18A91A^-LEU2 STU2-GFP-TRP1 TUB1-mApple-HIS3*  *MATa swe1Δ::LEU2 PDS1-18myc-LEU2 TUB1-GFP-URA3*  *MATa PDS1-18myc-LEU2 TUB1-GFP-LEU2*  *MATa swe1Δ::LEU2 POL12-13myc-Kan*  *MATa POL12-13myc-Kan*  *MATa cdc14-1 swe1Δ::LEU2 TUB1-GFP-LEU2*  *MATa swe1Δ::LEU2 net1Δ::Sphis5+ net1-6Cdk-TEV-myc9::TRP1 cdc14::CDC14-5GFP-TRP1*  *MATa spo12Δ::Kan SPC110-13myc-Sphis5+* | Lab stock  Lab stock  Lab stock  Lab stock  This study  This study  Lab stock  This study  Lab stock  Lab stock  Lab stock  This study  This study  This study  This study  This study  This study  This study  Toh-e lab  Lab stock  This study  This study  Lab stock  Lab stock  Lab stock  This study  This study  This study  This study  Lab stock  This study  This study  Lab stock  This study  This study  This study  Lab stock  This study  This study  This study  Lab stock  This study  This study  This study  Lab stock  This study  Lab stock  This study  This study  This study |
